# GelMA and Biomimetic Culture Allow the Engineering of Mineralized, Adipose, and Tumor Tissue Human Microenvironments for the Study of Advanced Prostate Cancer In Vitro and In Vivo

**DOI:** 10.1002/adhm.202201701

**Published:** 2023-02-21

**Authors:** Agathe Bessot, Jennifer Gunter, David Waugh, Judith A. Clements, Dietmar W. Hutmacher, Jacqui McGovern, Nathalie Bock

**Affiliations:** ^1^ School of Biomedical Sciences Faculty of Health, and Translational Research Institute (TRI) Queensland University of Technology (QUT) Brisbane QLD 4102 Australia; ^2^ Australian Prostate Cancer Research Centre ‐ Queensland (APCRC‐Q) QUT Brisbane QLD 4102 Australia; ^3^ School of Mechanical Medical and Process Engineering Engineering Faculty QUT Brisbane QLD 4000 Australia; ^4^ Centre for Biomedical Technologies QUT Brisbane QLD 4000 Australia; ^5^ Max Planck Queensland Centre Brisbane QLD 4059 Australia; ^6^ Centre for Genomics and Personalised Health QUT Brisbane QLD 4102 Australia

**Keywords:** 3D cancer models, adipocytes, bone tumor microenvironments, GelMA, humanized models, hydrogels, microtissues

## Abstract

Increasing evidence shows bone marrow (BM)‐adipocytes as a potentially important contributor in prostate cancer (PCa) bone metastases. However, a lack of relevant models has prevented the full understanding of the effects of human BM‐adipocytes in this microenvironment. It is hypothesized that the combination of tunable gelatin methacrylamide (GelMA)‐based hydrogels with the biomimetic culture of human cells would offer a versatile 3D platform to engineer human bone tumor microenvironments containing BM‐adipocytes. Human osteoprogenitors, adipocytes, and PCa cells are individually cultured in vitro in GelMA hydrogels, leading to mineralized, adipose, and PCa tumor 3D microtissues, respectively. Osteoblast mineralization and tumor spheroid formation are tailored by hydrogel stiffness with lower stiffnesses correlating with increased mineralization and tumor spheroid size. Upon coculture with tumor cells, BM‐adipocytes undergo morphological changes and delipidation, suggesting reciprocal interactions between the cell types. When brought in vivo, the mineralized and adipose microtissues successfully form a humanized fatty bone microenvironment, presenting, for the first time, with human adipocytes. Using this model, an increase in tumor burden is observed when human adipocytes are present, suggesting that adipocytes support early bone tumor growth. The advanced platform presented here combines natural aspects of the microenvironment with tunable properties useful for bone tumor research.

## Graphical abstract

The role of bone marrow adipocytes in advanced prostate cancer is still poorly defined, due to a lack of relevant models. Therefore, in this study, bioengineered human bone tumor platforms are developed using GelMA hydrogels. Mineralized, adipose, and prostate cancer 3D microtissues are generated in vitro, before being implanted in vivo to create an advanced humanized fatty bone tumor microenvironment.

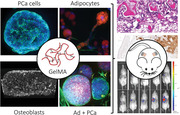

## 1 Introduction

Prostate cancer (PCa) is the second most commonly diagnosed cancer in men in the world.^[^ ^1^ ^]^ While PCa initially responds to androgen‐deprivation therapies, the development of castrate‐resistant PCa is inevitable and PCa thrives in the bones, the primary metastatic site.^[^ ^2^ ^]^ At this stage, the 5‐year survival rates drop from 56% to 3%.^[^ ^3^ ^]^ The tumor microenvironment significantly contributes to this poor outcome, via a complex interplay of the tumor cells with other microenvironment cells and interactions with the extracellular matrix (ECM). Cancer‐associated fibroblasts, vascular endothelial cells and multiple cell lineages present in this microenvironment modulate disease progression through vascularization, leading to disease recurrence and treatment resistance.^[^ ^4^ ^]^ While osteoblasts are key contributors in PCa bone metastases,^[^ ^5^ ^]^ the importance of bone marrow (BM)‐adipocytes in cancer is only emerging. It has been shown that adipocytes are important players in supporting tumorigenesis and metastasis, notably in myeloma, breast, and ovarian cancer, by providing a major source of prosurvival factors (cytokines and adipokines) and energy (free fatty acids) for cancer cell survival.^[^ ^6^ ^]^ Studies in the last ten years have started to show how PCa cells are capable of using cytokines provided by BM‐adipocytes allowing cancer to thrive in skeletal sites.^[^ ^7^ ^]^ However, the specific roles of BM‐adipocytes on PCa bone metastases and the timing of their contribution (early, late stage) are still poorly defined.

A critical step to better understand the specific roles of adipocytes in PCa bone metastases is the development of relevant models that better replicate the human bone tumor microenvironment. Although 2D models have provided important knowledge about cell crosstalk, they fail to adequately mimic the native microenvironment where cell behavior and phenotype are influenced by cell‐ECM and cell–cell interactions.^[^ ^8^, ^9^ ^]^ 3D models using engineered biomaterials better mimic naturally‐occurring ECM components and their 3D architecture, providing a more physiologically‐representative microenvironment for cells.^[^ ^9^, ^10^ ^]^ To mimic the complexities of native ECM in 3D models, various biomaterials have been used with various degrees of success.^[^ ^11^ ^]^ In cancer research, matrices of natural origin, including Matrigel, fibrin, collagen and gelatin are widely used.^[^ ^11^, ^12^ ^]^ Although Matrigel is considered the gold standard in mimicking the natural ECM, it presents intrinsic batch‐to‐batch variability and contains undefined elements as well as many fold higher growth factors content compared to native tissue, restricting data reproducibility and interpretation.^[^ ^13^ ^]^ To bridge the gap between synthetic and biological materials, semisynthetic matrices represent a good alternative, combining the advantages of natural ECM components and tunable properties, leading to higher reproducibility.^[^ ^14^ ^]^

In this study, gelatin methacrylamide (GelMA)‐based hydrogels were used as 3D cell culture systems for in vitro and in vivo use. This type of gel represents an appropriate tool to recreate the bone microenvironment as made of gelatin, a derivative of collagen, which constitutes ≈90% of the bone organic matrix.^[^ ^15^ ^]^ The hybrid structure of GelMA presents a combination of advantages with; 1) high biocompatibility due to its semi‐natural origin, and; 2) tunable mechanical properties due to its semi‐synthetic origin.^[^ ^16^, ^17^ ^]^ As a result, gel stiffness can be easily modified, using different polymer concentrations or crosslinking times, to match the mechanical properties of the different bone compartments; from hard bone (stiffnesses >35 kPa) to the marrow compartment (stiffnesses <2 kPa).^[^ ^18^ ^]^ GelMA is a well‐established hydrogel,^[^ ^19^, ^20^ ^]^ widely used to grow cells from the bone microenvironment, including bone marrow mesenchymal stem cells (BM‐MSCs),^[^ ^21^, ^22^ ^]^ osteoprogenitors,^[^ ^23^ ^]^ adipocytes^[^ ^21^, ^24^ ^]^ as well as cancer cells, such as prostate cancer cells,^[^ ^16^, ^25^ ^]^ and represent an excellent alternative to Matrigel and collagen‐based hydrogels, whose stiffnesses cannot easily be tuned to mimic specific microenvironments. In addition to being an appropriate tool for in vitro 3D platforms, GelMA hydrogels can also be used for in vivo studies as a biocompatible and biodegradable cell carrier, allowing human tissue formation in vivo,^[^ ^16^, ^26^ ^]^ and therefore represent an ideal candidate for both the in vitro and in vivo engineering of the human bone tumor microenvironment.

To study the role of the microenvironment on PCa bone metastases progression and therapy resistance, murine models have traditionally been used, yet they are unable to reconstitute the physiological or pathological processes in humans, hence humanized models are needed, better replicating species‐specific interactions.^[^ ^27^ ^]^ Our group developed humanized models to study the crosstalk between human cancer cells and humanized bone microenvironments in vivo, by developing ectopically‐ or orthotopically‐formed human ossicles in immunodeficient mice. Morphological features of bone were observed^[^ ^28^, ^29^ ^]^ and these models allowed the study of metastatic PCa and other malignancies.^[^ ^26^, ^28^, ^30^ ^]^ However, these models did not allow studying the interactions between human BM‐adipocytes and PCa cells within the humanized bone microenvironment as BM‐adipocytes were of murine origin.

In the present study, we hypothesized that GelMA‐based hydrogels would be a versatile candidate for engineering mineralized, adipose and tumor microtissues of human origin, for the in vitro and in vivo study of the bone tumor microenvironment. We encapsulated human cells in GelMA hydrogels of various stiffnesses, cultured with biomimetic culture media conducive to the engineering of different microtissue types. Mineralized microtissues were obtained using an innovative mineralizing media, and large 3D PCa tumors formed from single PCa cells. Tumor spheroid size was tailored according to GelMA stiffness. Adipose microtissues were obtained in vitro upon successful adipogenic differentiation from human mesenchymal or preadipocyte cells as early as two weeks. These models were further used in vivo and led to the first humanized fatty bone microenvironment comprising of a human adipocyte‐rich bone marrow. The fatty model was used to investigate the interactions between human cancer cells and adipocytes at an early colonization stage. By mimicking the local microenvironment of cancer cells invading the bone marrow, we showed that the presence of human adipocytes significantly enhanced tumor growth within the bone marrow microenvironment.

## 2 Results

### 2.1 Lower Stiffness GelMA and Biomimetic Culture Allow the Engineering of Human Osteoblast 3D Microtissues with Enhanced Mineralization

Biomechanical properties and biochemical cues are critical parameters to ensure the relevant engineering of mineralized 3D tissue models. Stiffness in particular is a large contributor to cellular functions.^[^ ^31^ ^]^ Here, primary osteoprogenitors were embedded in GelMA hydrogels of 5–10% (w/v) polymer concentrations as a mean to study stiffnesses ranging within an order of magnitude, according to previously published literature and our own mechanical data.^[^ ^32^ ^]^ Crosslinking time and intensity can be varied to tune stiffness, yet we chose to keep those parameters constant and focus on polymer concentration as a well‐characterized tailorable parameter in GelMA hydrogels. To determine the effect of concentration on the mechanical properties of GelMA hydrogels, compression tests were performed on samples containing different GelMA concentrations prior to in vitro studies. We observed that an increase of GelMA concentration from 4% to 10% (w/v) led to an increase in stiffness from 2.5 kPa ± 0.4, similar to the bone marrow compartment, to 40.2 kPa ± 3.2 (mean ± SD (standard deviation), Figure S1, Supporting Information), similar to the hard bone compartment. The correlation between hydrogel stiffness and polymer concentration demonstrates the mechanical tuneability of GelMA hydrogels and relevance to mimic bone‐like microenvironments, in agreement with previous studies.^[^ ^32^ ^]^

**Figure 1:**
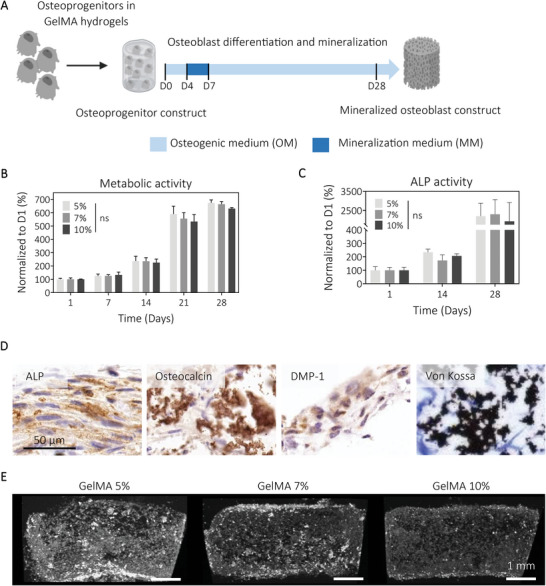
Engineering mineralized microtissues using tunable GelMA hydrogel and biomimetic culture. A) Schematic of the experimental design. Human osteoprogenitors were embedded in GelMA 5%, 7%, and 10% (w/v) hydrogels and cultured in osteogenic (OM) for up to four weeks with a short course in mineralization media (MM, 3 days). B) Metabolic activity of the osteoblast constructs showed significant increase in activity over time, with no significant differences between conditions. C) Alkaline phosphatase activity normalized to DNA content showed an increase in activity over time, similar between conditions. D) Representative images of immunohistochemistry (IHC) analyzes from GelMA 5% hydrogels condition for ALP, osteocalcin, and dentin matrix protein 1 (DMP‐1) and Von Kossa staining confirmed osteoblastic differentiation, ECM deposition and mineral formation after four weeks of culture in vitro. E) µCT representative images of osteoblast mineralization (minerals in white, scale bar = 1 mm) after 28 days of culture showed a higher mineral deposition in the low GelMA concentration. Means ± SEM, *n* = 3, univariate general linear model, ns = no statistical difference.

We examined the effect of hydrogel stiffness on osteoblast viability and mineralization with the hypothesis that higher stiffnesses would correlate with reduced mineralization. The human compact bone and bone marrow (BM) endosteal compartment present with high stiffnesses (>35 kPa);^[^ ^33^ ^]^ however, when developing 3D culture models, studies have shown that higher viability and mineralization from osteoprogenitors may be obtained with lower stiffnesses, likely due to enhanced remodeling capacity.^[^ ^22^ ^]^ Therefore, we tested three stiffnesses using 5%, 7%, and 10% (w/v) GelMA, with respective stiffnesses of 6.3, 16.3, and 40.2 kPa (Figure S1, Supporting Information). The human osteoblast tissue constructs (hOTC) were cultured up to four weeks in osteogenic medium (OM) in vitro to induce osteogenic differentiation, with three days in mineralization medium (MM) to enhance nanoscale biomineralization (**Figure** **1**A). The mineralization medium, initially introduced by Thrivikraman et al.,^[^ ^34^ ^]^ contains excess calcium (Ca^2+^) and phosphate (PO_4_^3−^) ions to promote protein‐induced mineralization within a collagen‐based hydrogel.^[^ ^34^ ^]^ We hypothesized that this would be true for GelMA and used MM to boost mineralization compared to traditional osteogenic media (OM), which can take up to 13 weeks of culture to provide sufficient biomineralization in GelMA and other 3D models.^[^ ^35^, ^36^, ^37^, ^38^ ^]^ Therefore, before examining the effect of hydrogel stiffness on mineralized tissue formation, we first confirmed the use of MM on GelMA 5% hydrogels. From Figure S2 (Supporting Information), it was shown that the short use of MM on hOTC was superior to traditional OM, depicting enhanced biomineralization using this novel biomimetic culture. To determine if cell viability could be affected by hydrogel stiffness, we determined the percentage of living cells using FDA/PI staining. On day 1, cell viability was high (≈90% living cells) with no significant differences between conditions (Figure S3A, Supporting Information). After MM culture (day 4–7) and further culture in OM, determination of cell viability using FDA/PI staining was not possible due to minerals blocking detection of the fluorescent signal via microscopy imaging (Figure S3A, Supporting Information). Metabolic activity assessed weekly increased over time, with no significant differences between GelMA concentrations (Figure 1B). These data showed that the polymer concentrations tested within a stiffness range of 6.3–40.2 kPa were not significantly affecting cell viability. We then evaluated whether hydrogel stiffness could influence osteoblast differentiation as previous studies have shown that lineage specification could be affected by biophysical aspects, including matrix stiffness and amounts of RGD‐specific adhesion sites.^[^ ^39^ ^]^ Alkaline phosphatase (ALP) is an early marker of osteoblasts involved in the initiation of mineralization of the ECM^[^ ^40^ ^]^ and thus was measured here up to 28 days of culture. In Figure 1C, no significant ALP differences were observed between GelMA 5%, 7%, and 10% (w/v) regardless of time. Osteoblast differentiation was confirmed using immunohistochemistry (IHC) staining of ALP, osteocalcin (mature osteoblast marker), and dentin matrix protein 1 (DMP‐1, osteocyte marker). No significant differences between conditions were observed from these stainings (Figure 1D; Figure S4A, Supporting Information) except for DMP‐1 which was not detected in GelMA 10% constructs (Figure S4A, Supporting Information). Mineral deposition was evaluated chemically and by micro‐computed tomography (µCT) analyzes. As shown in Figure 1D and Figure S4A (Supporting Information) (Von Kossa staining), all conditions presented mineral deposition after four weeks of culture, with higher mineral levels detected in lower stiffness constructs (Figure S4B, Supporting Information), in line with our hypothesis. However, differences between conditions were not significant, likely due to variability between biological replicates. Data from µCT confirmed that mineral deposition was inversely correlated to stiffness (Figure 1E) and showed no significant difference between conditions (Figure S4C, Supporting Information). Osteoimage staining confirmed the presence and formation of bone mineral (hydroxyapatite) over time in all conditions, as shown in Figure S4D (Supporting Information). These findings suggest that lower stiffness hydrogels enabled enhanced osteoblast differentiation and increased mineralization. This is line with the study from Celikkin et al. using BM‐mesenchymal stem cells (BM‐MSCs) seeded on GelMA scaffolds.^[^ ^22^ ^]^

### 2.2 Low (2–12 kPa) Stiffness GelMA Hydrogels Can Reproducibly Engineer Tumor Microtissues Containing Human Prostate Cancer Spheroids

**Figure 2:**
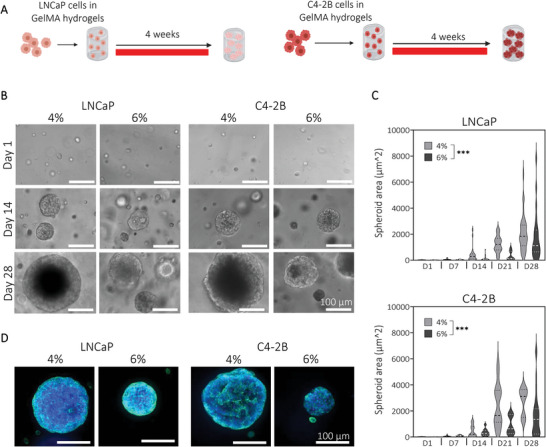
Engineering prostate cancer microtissue using tunable GelMA hydrogels. A) Schematic of experimental design. PCa cell lines C4‐2B and LNCaP were embedded in GelMA 4% and 6% (w/v) hydrogels and cultured into spheroids for up to four weeks. B) Phase‐contrast microscopy images showed the formation of spheroids in both prostate cancer cell lines from day 1 to day 28 (scale bar = 100 µm). C) Spheroid area quantification indicated smaller spheroid formation in the high stiffness compared to the low stiffness in both cell types (Violin plots, line at median and quartile, *n* = 3, *N* = 120 spheroids per cell type, univariate general linear model, ***P < 0.001). D) Immunofluorescence staining using DAPI (blue, nuclei) and Phalloidin (green, actin filaments) showed smaller spheroids in the highest stiffness compared to the lowest stiffness after 28 days of culture (scale bar = 100 µm).

Gel culture is a common way to obtain tumor spheroids akin to tumors found in vivo in soft tissues (breast, prostate, brain, liver),^[^ ^41^ ^]^ yet cancer cells are strongly influenced by the stiffness of the environment.^[^ ^42^, ^43^ ^]^ To mimic prostate cancer tumors in vitro, we thus sought to identify an appropriate hydrogel stiffness range that would enable reproducible spheroid formation. We tested 2.5 kPa ± 0.3 and 11.7 kPa ± 0.8 (Figure S1, Supporting Information), corresponding respectively to 4% and 6% (w/v) GelMA, similar to bone marrow adipose tissue and prostate tissue, yet with over a 4‐fold difference, enabling mechanical tuning.^[^ ^33^, ^44^ ^]^ We hypothesized that the lower stiffness would lead to enhanced PCa growth, yet maintain organoid spherical shape, enabling reproducibility. Metastatic prostate cancer cells LNCaP and C4‐2B were embedded in the selected GelMA hydrogels and cultured for four weeks to form spheroids (**Figure** **2**A). Metabolic activity was monitored weekly, showing an increase from day 1 to day 21 and a decrease after 3 weeks of culture in both cell types, with no significant difference between conditions (Figure S5, Supporting Information). Cell viability remained high during the culture period, showing viable spheroids with necrotic centers in each condition (Figure S3C, Supporting Information), mimicking the cell heterogeneity found in microregions of tumors.^[^ ^43^, ^45^ ^]^ Spheroid formation from both LNCaP and C4‐2B cells was observed using phase‐contrast microscopy images weekly. As displayed in Figure 2B, spheroid formation started during the first weeks of culture in both cell types. Spheroid size, measured using Image J software, showed smaller spheroids in the highest stiffness (area average of 1770 µm^2^ for LNCaP and 1681 µm^2^ for C4‐2B spheroids at day 28) compared to the lowest (2052 and 2756 µm^2^ respectively, Figure 2C), as confirmed by DAPI/Phalloidin staining on samples at day 28 (Figure 2D), consistent with other studies^[^ ^46^, ^47^, ^48^ ^]^ and in line with our hypothesis. It could be concluded that low (2–12 kPa) stiffness GelMA hydrogels were conducive to reproducible prostate cancer tumor formation with physiologically relevant morphologies and processes (necrotic core formation).

### 2.3 Fat and Bone Marrow‐Derived Progenitors Cultured in Very Low Stiffness (<3 kPa) GelMA Can Both Be Differentiated in Adipocytes, Leading to Human Adipose Microtissues

**Figure 3:**
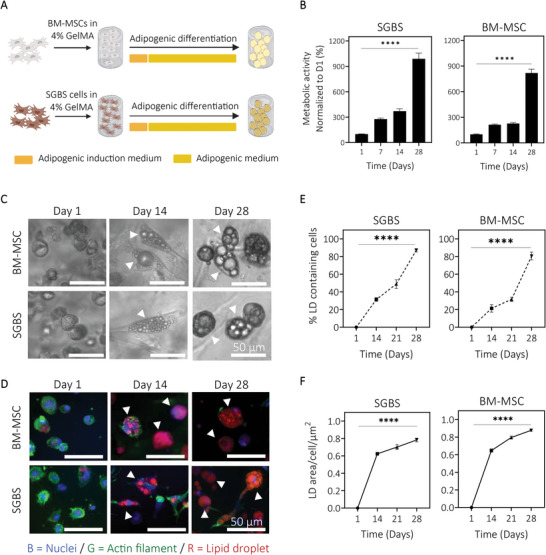
Engineering adipose microtissues using GelMA hydrogels. A) Schematic of the experimental design. Human bone marrow (BM)‐MSCs and fat derived preadipocyte SGBS cells were embedded in GelMA 4% (w/v) hydrogels and cultured under adipogenic differentiation for up to four weeks. B) Metabolic activity showed significant increase over time, for both constructs with BM‐MSCs and SGBS cells. C) Phase‐contrast microscopy images and D) immunofluorescence staining using DAPI (Blue, Nuclei), Phalloidin (Green, Actin) and Nile Red (Red, Lipid droplets) showed lipid droplets formation in both cell types on days 1, 14, and 28 (white arrows, scale bar = 50 µm). E) Quantification of proportion of cells containing lipid droplets (LD) and F) lipid droplet area per cell indicated significant increase in lipid droplets content over time (*N* = 213 cells analyzed per condition). Means ± SEM, *n* = 3, univariate general linear model, ****P < 0.0001.

With the demonstration of GelMA as a useful platform to obtain mineralized osteoblast 3D microtissues (2.1) and reproducible tumor spheroids (2.2), the next question was to assess GelMA's capacity to provide an in situ differentiated human adipose microtissue model. Bone‐marrow MSCs (BM‐MSCs) are the natural choice of cells to obtain BM‐adipocytes, yet they are costly and only present in small quantities in the bone marrow. While other MSC sources could be used, it is now recognized that fat‐derived adipocytes present differences compared to BM‐adipocytes,^[^ ^49^, ^50^ ^]^ although it is unclear if this affects crosstalk with cancer cells. As a result, we chose to do a comparative study between BM‐MSCs and Simpson–Golabi–Behmel syndrome (SGBS) preadipocytes, that are fat‐derived. By growing the two human precursor cell types in GelMA hydrogels, we hypothesized that no significant gene and morphological differences would be observed upon adipogenic differentiation, and that GelMA would successfully support adipogenesis in both cases. GelMA 4% (w/v) hydrogel was selected, corresponding to 2.5 kPa (Figure S1, Supporting Information) mimicking the low stiffness of native bone marrow adipose tissue, ranging below 3 kPa.^[^ ^33^ ^]^ Human BM‐MSCs and SGBS cells were embedded in the hydrogels and differentiated into adipocytes for up to four weeks in vitro (**Figure** **3**A). Before comparing BM‐MSCs to SGBS adipogenesis, preliminary studies were conducted to validate that adipogenic medium in both cell types was necessary to adipogenesis, and none of the cell types displayed lipid droplets in the absence of adipogenic media (Figure S6, Supporting Information). The resulting human adipose tissue constructs showed high viability over time, as observed by live/dead staining quantification (Figure S3B, Supporting Information). BM‐MSCs and SGBS cells showed a significant increase in metabolic activity from day 1 to day 28 (Figure 3B). Adipogenic differentiation was assessed after two and four weeks of culture. Intracellular accumulation of lipid droplets (LD) was detected using phase‐contrast microscopy images, suggesting adipogenesis of both BM‐MSCs and SGBS cells under adipogenic differentiation (Figure 3C, white arrows). Accumulation of LD was confirmed using Nile Red/DAPI/Phalloidin staining on samples at days 1, 14, and 28 (Figure 3D). As displayed in Figure 3D, cell morphology changed over time. While on day 1 cells were small and round, they started to show an elongated morphology after two weeks of culture before becoming large round cells, typical of adipocyte morphology. The proportion of LD‐containing cells increased over time with more than 80% after 28 days of differentiation for both cell types (Figure 3E). LD surface showed an increase over time, especially from day 1 to day 14 (Figure 3F). Next, Figure S7A (Supporting Information) showed that early adipogenic marker FABP4, lipogenic markers FASN, PLIN, GLUT4, and LIPE and secreted marker ADIPOQ had a similar expression profile in BM‐MSC and SGBS cells after 14 and 28 days of differentiation. Early adipogenic markers tended to increase over time, consistent with adipocyte maturation,^[^ ^51^ ^]^ although no significant differences were observed between day 14 and day 28 (Figure S7B, Supporting Information). However, differences in expression levels of early markers PPAR*γ* and C∖EBP*α* were observed between both cell types. Specifically, expression of PPAR*γ* and C∖EBP*α* (Figure S7B, Supporting Information) tended to increase over time in BM‐MSC culture, while it decreased or stabilized in SGBS cells. However, these differences were not statistically significant. These findings show that the adipogenic human progenitors used here, namely BM‐MSCs and SGBS, could be both cultured and differentiated in low stiffness GelMA hydrogels for up to four weeks. Both generated a viable adipose microtissue in vitro, with minimal differences between the cell types, enhancing model versatility according to progenitor access.

### 2.4 Low Stiffness GelMA (2.5 kPa) Can Support the Coculture of Human Adipocytes and Human Prostate Cancer Spheroids with Effective Adipocyte Dedifferentiation

**Figure 4:**
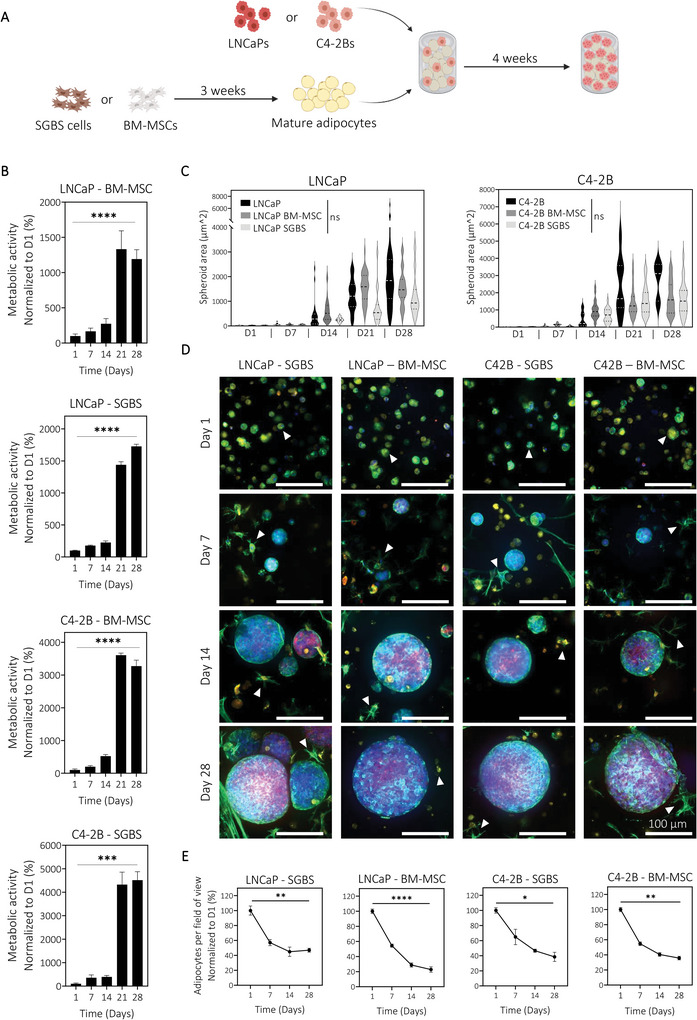
Engineering a human adipocyte‐prostate cancer cell direct coculture. A) Schematic showing the experimental timeline of direct coculture between human adipocytes and prostate cancer cell lines LNCaP and C4‐2B, embedded in 4% (w/v) GelMA hydrogels. B) Metabolic activity showed significant increase in activity over time, for each coculture (means ± SEM, *n* = 3, ***P < 0.001, ****P < 0.0001*)*. C) Spheroid area quantification showed similar spheroid formation in the presence of SGBS‐ and BM‐MSC‐derived adipocytes compared to single culture (Violin plots, line at median and quartile, *N* = 114 spheroids analyzed per condition). D) Immunofluorescence staining using DAPI (blue, nuclei) and Phalloidin (green, actin filaments) indicated the formation of spheroids in both prostate cancer cell lines (red, RFP) cocultured with human adipocytes (yellow, DiR, white arrows), with changes in adipocytes morphology after one week of coculture (scale bar = 100 µm). E) Quantification of number of adipocytes detected per field of view (FOV) from immunofluorescence staining images showed a significant decrease of adipocytes after one week of coculture (means ± SEM, *n* = 3, *N* = 8 FOV per condition, univariate general linear model, *P < 0.05, **P < 0.01, ****P < 0.0001).

The previous models successfully demonstrated the growth, differentiation, and mineralization of various GelMA constructs loaded with individual human cell types. Next, we created an adipocyte‐PCa cell coculture model to explore complex adipocyte‐PCa cell reciprocal interactions. As it is known that adipocytes dedifferentiate in the presence of cancer cells,^[^ ^7^, ^52^, ^53^, ^54^ ^]^ our hypothesis was that a similar effect would be observed in a 3D in vitro coculture. Mature adipocytes, generated from BM‐MSCs and SGBS cells and labeled with a far‐red dye (DiR) were embedded in 4% (w/v) GelMA hydrogels with RFP‐labeled LNCaP or C4‐2B cells and cultured for up to four weeks in vitro (**Figure** **4**A). Prior to coculturing adipocytes and PCa cells together, coculture medium was validated on single cell cultures for both cell types to ensure its suitability for spheroid formation and mature adipocytes culture (Figure S8, Supporting Information). Upon direct coculture, metabolic activity showed a significant increase over time in each condition, especially between day 14 and day 21 (Figure 4B). Spheroid formation and size were unaffected by the presence of adipocytes (Figure S9, Supporting Information; Figure 4C). C4‐2B cells cocultured with SGBS‐ and BM‐MSC‐derived adipocytes showed a higher increase in spheroid size during the first 2 weeks of culture compared to C4‐2B cells alone. However, over the four weeks of coculture, differences between conditions were not statistically significant. Importantly, immunofluorescence staining showed changes in adipocyte morphology over time (Figure 4D, white arrows); round cells shrank and became fibroblastic after one week of coculture, which is also seen in cancer‐associated adipocytes and highlight dedifferentiation, in line with our hypothesis.^[^ ^52^, ^53^ ^]^ In addition, a significant decrease in the number of fat cells was observed, especially after one week of coculture, with a 40% decrease compared to day 1 (Figure 4E). This was seen by other groups after one week of coculture with other cancer types, both in vitro and in vivo.^[^ ^53^, ^54^ ^]^ These results strongly display the power of the coculture platform presented here in recapitulating physiological processes undergone by adipocytes in the presence of cancer cells, similarly to known in vivo tumor contexts.

### 2.5 Engineering a Humanized Fatty Bone Microenvironment In Vivo Using Mineralized and Adipose‐Like GelMA Microtissues

**Figure 5:**
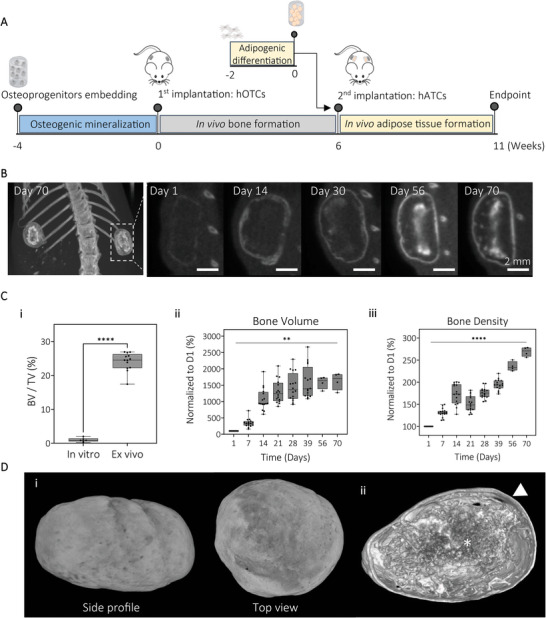
Humanized bone tissue formation in vivo using mineralized GelMA microtissues. A) Schematic of experimental design. Human osteoprogenitors were embedded in GelMA 5% (w/v) hydrogels and cultured for four weeks to form a mineralized tissue before subcutaneous implantation in mice. After six weeks of in vivo bone formation, adipose tissue constructs (hATCs) were implanted within the same subcutaneous pocket as human osteoblast tissue constructs (hOTC) and cultured for five weeks in vivo. B) Representative *µ*CT images from day 1 to day 70 post‐implantation of the hOTCs indicated successful bone formation in vivo (scale bar = 2 mm). C) *µ*CT quantification of i) bone volume fraction before and after in vivo culture and ii) bone volume and iii) bone density of the hOTCs over time (box and whisker plot (min‐to‐max), line at median, *n* = 16, univariate general linear model, **P < 0.01, ****P < 0.0001). D) Mineralized tissue is shown in representative *µ*CT reconstruction of hOTCs after 11 weeks in vivo i) external view and ii) cross‐section view showing cortical shell (white arrow) with trabecular network inside the organ (white star).

Based on our in vitro data, we selected the mineralized tissue model presented in 2.1 (GelMA 5% (w/v)) for the establishment of a humanized bone niche. After four weeks culture in vitro, the mineralized constructs were implanted subcutaneously in the flank of NSG mice. Upon in vivo bone tissue formation (6 weeks), the adipose constructs, cultured prior for three weeks in vitro under adipogenic differentiation, were implanted within the same subcutaneous pocket as hOTC to create a humanized fatty bone microenvironment (**Figure** **5**A). Mineralization was detected in all microtissues, as soon as day 1 in vivo (Figure 5B). Mineralization was initially restricted to the gel boundaries, however, after 30 days in vivo, it started to occur inside the constructs. The µCT quantification of the mineralized tissue volumetric density in the microtissues before and after in vivo culture indicated that volumetric density significantly increased after 11 weeks in vivo (Figure 5C‐i). While bone volume increased drastically during the first two weeks and started to stabilize after three weeks in vivo (Figure 5C‐ii), bone density increased constantly during the 11 weeks (Figure 5C‐iii). Data reconstruction showed that tissue kept their initial cylindrical shape and presented a highly mineralized cortical shell after 11 weeks in vivo (Figure 5D‐i). Cross‐section of microtissues showed a cortical shell surrounding a porous network (Figure 5D‐ii), similar to trabecular bone.

**Figure 6:**
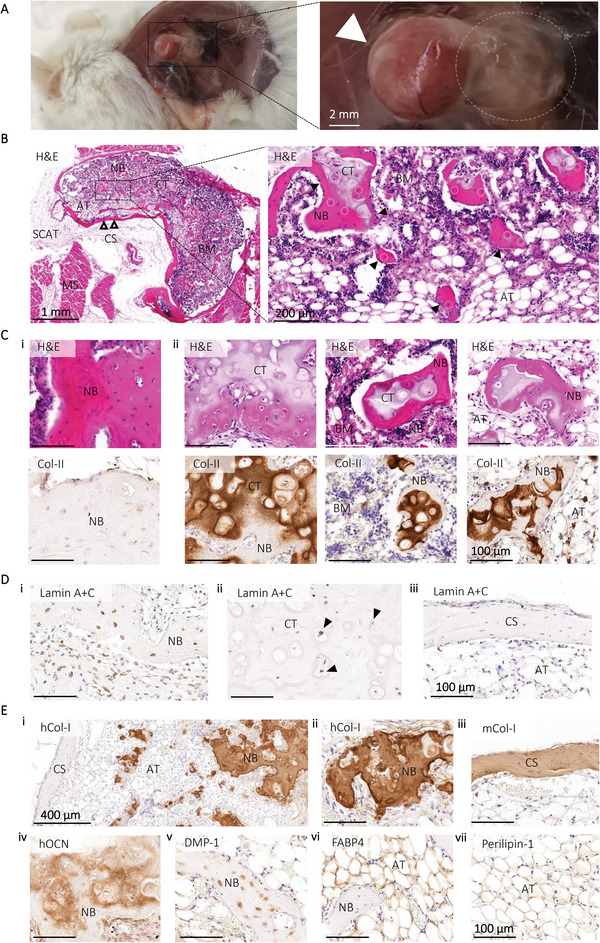
Characterization of the humanized bone model. A) Representative images of humanized bone‐like tissue after 11 weeks in vivo (white arrow) with adipocyte‐loaded hydrogel after five weeks in vivo (white circle). B) Representative H&E images at low and high magnification showed the morphology of the bone ossicle (live osteocytes shown with white circles; bone‐lining osteoblasts with black arrows). C) Images from H&E staining indicated that new bone formation occurred via i) direct intramembranous and ii) endochondral ossification. Morphological observations were confirmed using IHC for collagen II (brown stain) which showed i) collagen II‐negative areas of new bone and ii) cartilage‐like structures and bone positive for collagen‐II. D) IHC analyzes for human Lamin A+C (brown stain) confirmed the presence of human cells in the i) bone and ii) cartilaginous tissue. iii) The outer ossicle was negative for this human‐specific marker. E) IHC for i,ii) human‐derived collagen type I (brown staining) showed outer surface of the ossicle negative for human collagen I, and the presence of positive staining inside the construct, while iii) murine‐derived collagen type I showed positive staining in the outer surface. iv) hOCN showed positive staining within the constructs, as well as v) DMP‐1 which confirmed the presence of osteocytes. vi) FABP4 and vii) Perilipin‐1 confirmed presence of adipocytes within the humanized bone construct. NB, new bone; AT, adipose tissue; CT, cartilaginous tissue; BM, bone marrow; CS, cortical shell; SCAT, subcutaneous adipose tissue; MS, muscle.

After in vivo culture (**Figure** **6**A), H&E staining of the center of the explant indicated the presence of a mature bone tissue made of a cortical‐like bone outer layer inclosing trabecular structures, presenting osteocytes and bone‐lining osteoblasts (Figure 6B). Other tissue types were observed in the construct, including bone marrow, cartilage‐like tissue, and adipose tissue. Some residual GelMA hydrogel could be sporadically detected (Figure S10, Supporting Information). The presence of cells cluster surrounding isolated bone spicules was observed (Figure 6C‐i). These structures were negative for collagen type II (Col‐II), showing intramembranous bone formation without a cartilage intermediary. However, cartilage‐like structures positive for Col‐II could also be detected, mainly in the center of the hOTCs (Figure 6C‐ii). Most of the bone spicules observed within the constructs displayed cartilaginous‐like tissue surrounded by calcified bone matrix, and were Col‐II‐positive, positively indicating that the different stages of endochondral ossification were replicated within the microtissues (Figure 6C‐ii). Masson's trichrome and Safranin O/Fast Green stainings confirmed the presence of newly formed bone spicules from collagen deposition and cartilage tissue (Figure S11, Supporting Information). IHC analyzes with human‐specific antibodies showed human cells throughout the inner cancellous bone. Lamin A+C staining indicated human cells were present within the bone tissue (Figure 6D‐i,ii). However, cells embedded within the cortical shell were negative for Lamin A+C (Figure 6D‐iii). This likely indicates that the cortical shell is formed from host cells. This hypothesis was confirmed by human‐derived type I collagen (hCol‐I) and murine Col‐I (mCol‐I) stainings. hCol‐I, an early marker of osteoblast activity and bone formation,^[^ ^55^ ^]^ was detected inside the bone construct but was negative in the cortical shell (Figure 6E‐i,ii). The presence of large areas positive for this marker indicated that the implanted cells produced a human‐derived ECM within the murine host. Murine‐derived type I collagen (mCol‐I) was also detected within the constructs, especially in the cortical shell, which indicates the hybrid bone tissue formation (Figure 6E‐iii). Human osteocalcin (hOCN), determined using a human‐specific antibody, was expressed in the hOTC (Figure 6E‐iv). Positive staining of human‐specific bone matrix proteins was found mostly in the center of the constructs, whereas the outer cortical layer was negative. These stainings showed that the hOTCs contained an important proportion of human‐specific bone components, both at the cell and ECM levels, whereas cortical shell and bone marrow content were derived from the host. Cells embedded within bone structures were positive for the late‐stage bone marker dentin‐matrix protein 1 (DMP‐1), indicating their osteocyte lineage (Figure 6E‐v). Adipose tissue was detected within the bone constructs and confirmed using FABP4, an early marker of adipogenesis, and Perilipin‐1, a lipid droplet‐associated protein, staining (Figure 6E‐vi,vii).

In adipose constructs, cells expressed FABP4, confirming adipocyte lineage (Figure S12A, Supporting Information, black arrows). Furthermore, the constructs were surrounded by an adipose tissue where some adipocytes were positive to LaminA+C staining, which indicated a hybrid adipose tissue from human and murine origin (Figure S12B, Supporting Information, black arrows). These data indicate that, for the first time, a humanized fatty bone microenvironment comprising of a human adipocyte‐rich human bone marrow was formed using the GelMA‐based approach.

### 2.6 Human Adipocytes from the Humanized Fatty Bone Microenvironment Contribute to Human Prostate Cancer Tumor Growth In Vivo

**Figure 7:**
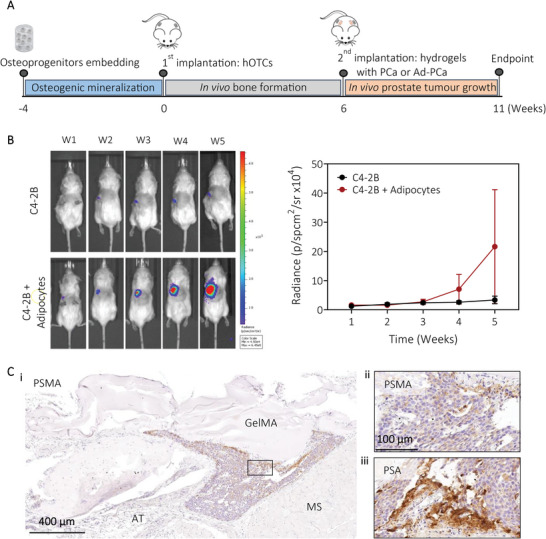
Human adipocytes from the humanized fatty bone microenvironment contribute to human prostate cancer tumor growth in vivo. A) Schematic of the experimental design. Human osteoprogenitors were embedded in GelMA 5% (w/v) hydrogels and cultured for four weeks to form a mineralized tissue before subcutaneous implantation in mice. After six weeks of in vivo bone formation, C4‐2B cells embedded in GelMA 4% (w/v) hydrogels with or without SGBS‐derived adipocytes were implanted within the same subcutaneous pocket as the humanized bone and cultured for five weeks in vivo. B) Representative bioluminescence‐photographic overlay images and quantification showed C4‐2Bs growth within the humanized bone microenvironment over time, detected by in vivo BLI (mean ± SEM, *n* = 2 for C4‐2B and control, *n* = 4 for C4‐2B + adipocytes). C) IHC for i,ii) PSMA and iii) PSA showed cancer cells growth. AT, adipose tissue; MS, muscle.

As it is known that adipocytes contribute energy to support tumorigenesis and metastasis in several cancers, we sought to use the humanized fatty bone microenvironment developed here to assess whether human adipocytes have a similar effect on prostate cancer cells. The humanized bone was formed in vivo for six weeks using the mineralized GelMA construct (2.1, 5% w/v) prior to adding separate GelMA hydrogels containing luciferase‐C4‐2B cells, with or without mature SGBS‐derived adipocytes (**Figure** **7**A). We hypothesized that tumor burden would be enhanced in the presence of human adipocytes. In vivo bioluminescence indicated that C4‐2B cells were able to grow in vivo in this model and were enhanced by the presence of the adipocytes (Figure 7B). IHC analyzes of the combined constructs showed C4‐2B tumor formation (<1 mm^2^) and surrounded by adipose tissue, when cells were cocultured with SGBS‐derived adipocytes, confirmed by the prostate cancer specific markers PSA and PSMA staining (Figure 7C). By mimicking the local microenvironment of cancer cells invading the bone microenvironment, we showed for the first time, how the presence of human adipocytes significantly enhanced tumor growth within the humanized bone tumor microenvironment.

## 3 Discussion

Tumor engineering has opened a new perspective for in vitro and in vivo disease modeling and drug testing by better recapitulating parts of the native in vivo environment.^[^ ^56^, ^57^ ^]^ It is now well‐understood that tumor 3D modeling more accurately reflects tumor biology, as the extracellular matrix surrounding a tumor affects cell behavior, phenotype and invasiveness.^[^ ^42^, ^48^ ^]^ Increasing evidence has demonstrated the importance of choosing relevant biomaterials and stiffnesses to create 3D models.^[^ ^11^, ^31^ ^]^ Here, we hypothesized that GelMA‐based hydrogels would be a more tunable semisynthetic alternative to Matrigel, and other purely synthetic hydrogels, to create mineralized, adipose and tumor microtissues both in vitro and in vivo, and therefore develop the first humanized bone tumor models containing human BM‐adipocytes.

In our study, we demonstrated that increasing GelMA concentrations led to a significant increase in hydrogel stiffness. Gel stiffness impacts its structural parameters, including pore size, number of attachment sites and swelling ratio,^[^ ^22^, ^58^, ^59^, ^60^ ^]^ which can influence molecule diffusion within the gel, nutrient access, as well as cell attachment, viability, and phenotype.^[^ ^42^, ^48^, ^58^ ^]^ While previous studies showed better results in differentiating and maintaining bone cell morphology and phenotype when seeded on rigid matrices (>130 kPa),^[^ ^61^, ^62^, ^63^ ^]^ we demonstrated that embedding osteoprogenitors in a soft hydrogel (<20 kPa) led to better osteogenic differentiation and higher mineralization compared to stiff hydrogels (>35 kPa). As shown previously by our group and others, although macromolecules can diffuse within GelMA hydrogels of 0.5 kPa to >100 kPa, the diffusion rate, permeability and pore size are correlated to the polymer concentration, and so hydrogel stiffness.^[^ ^59^, ^64^, ^65^ ^]^ Thus, GelMA hydrogels presenting a stiffness lower than 20 kPa not only present better cell adhesion compared to higher stiffnesses but also a higher porosity allowing better distribution of calcium and phosphate ions within the hydrogel, leading to higher mineralization through the 3D network.^[^ ^22^, ^39^, ^66^ ^]^ We also demonstrated that GelMA hydrogel stiffness significantly affected PCa cell spheroid formation, where the lowest stiffness induced the largest spheroid formation, in both LNCaP and C4‐2B cell lines, without affecting cell viability. While pore size from GelMA hydrogels presenting a stiffness between 2 and 11 kPa ranges between 370 ± 60 and 250 ± 65 µm,^[^ ^22^ ^]^ allowing diffusion of macromolecules, adaptation of tumor spheroid growth to the compressive stress induced by matrix stiffness has been demonstrated, where low stiffnesses allowed bigger spheroid formation.^[^ ^67^ ^]^ In our study, we also observed a decrease of metabolic activity after 3 weeks of culture in GelMA hydrogels, which is expected when spheroids reach a diameter higher than 100 µm because of limited nutrient diffusion to the centre of the spheroid and therefore the formation of a necrotic core.^[^ ^43^, ^68^ ^]^

To study bone marrow adipose tissue, a variety of in vitro models have been used.^[^ ^69^ ^]^ However, even though in vitro 3D models better mimic the native tissue compared to 2D models, very few 3D models of bone marrow adipose tissue have been developed yet. While silk scaffolds have proven to be a relevant material for this purpose, leading to differentiation of BM‐MSC into functional adipocytes and creating a platform for myeloma study, yet this model lacks softness mimicry of bone marrow tissue.^[^ ^53^ ^]^ Recently, our group proved the feasibility of culturing and differentiating both white and bone marrow adipocytes progenitors (SGBS cells and BM‐MSCs respectively) within a soft matrix to create a more relevant adipose model that better mimics the native mechanical properties of fat tissue.^[^ ^21^ ^]^ Here, in addition to mimicking the softness of the bone marrow, we were able to differentiate BM‐MSCs and SGBS cells into mature and functional adipocytes, characterized by lipid droplet accumulation and adipogenic marker expression. Although SGBS cells were isolated from white adipose tissue, presenting significant differences in phenotype and functions compared to bone marrow adipose tissue, white and bone marrow adipocytes presented similarities in gene expression, as seen in our study and shown by other groups.^[^ ^70^ ^]^ In addition, as demonstrated by Menssen et al., BM‐MSCs differentiated into adipocytes in vitro showed similar adipogenic gene expression compared to native fat tissue after only 17 days.^[^ ^71^ ^]^ This confirmed that our bioengineered adipose tissue is versatile (preadipocytes from fat and BM could be used) and relatively quick to establish (2 weeks).

Additionally, we demonstrated that GelMA hydrogels were suitable tools to coculture human adipocytes with human PCa cells within 3D ECM microenvironments. While BM‐adipocytes‐PCa cell reciprocal interactions have been described in 2D cultures, showing lipid translocation between adipocytes and PCa cells and increased tumor invasion,^[^ ^72^, ^73^ ^]^ 3D models remain rare. Herroon et al. described the first 3D hydrogels‐based coculture of BM‐adipocytes with PCa cells using a collagen I matrix.^[^ ^74^ ^]^ Although this model allowed the study of BM‐adipocytes effects on PCa cells migration, the use of murine BM‐adipocytes failed to replicate species‐specific interactions. Therefore, to our knowledge, the model presented in our study is the first human hydrogel‐based 3D coculture model that contains human adipocytes and prostate cancer cells. It allowed direct human adipocytes‐PCa cells coculture within a soft matrix mimicking the bone marrow tissue. The changes in adipocyte behavior and phenotype have already been observed by other groups when cocultured with cancer cells, including prostate cancer cells.^[^ ^7^, ^52^, ^53^, ^54^ ^]^ Specifically, cancer cells were shown to first enhance adipogenic differentiation, leading to an increase in number of mature adipocytes, before adipocyte delipidation to fuel cancer cell invasion. At this stage, adipocytes presented a fibroblastic morphology and started to shrink, giving space for cancer cells to grow. This two‐step process was also described in vivo, where bone marrow presents an increase in fat content during early stage of bone marrow metastasis before showing a drastic decrease of fat content.^[^ ^54^, ^75^ ^]^ Hence, the coculture model presented here is a relevant in vitro tool to study the crosstalk between human BM‐adipocytes and PCa cells in the context of early stage bone tumor.

After optimization of our in vitro constructs, we hypothesized that these constructs would be suitable to create a relevant humanized bone tumor microtissues in vivo for the study of early stage bone tumor formation. Humanized models allow to overcome limitations observed in traditional bone metastasis xenograft models by developing a humanized bone microenvironment within a murine host. Humanization of in vivo models started a decade ago with implantation of the first tissue‐engineered bone models, made from sponges seeded with human osteoblasts or BM‐MSCs, as a target for human cancer cells into rodents.^[^ ^76^, ^77^ ^]^ Even if these models allowed human tumor growth, the resulting bone organ was lacking of a functional bone marrow niche and relevant physiological features. Moreover, no information about the species origin of the cells composing the newly formed bone tissue was provided. In past studies, our group demonstrated our capacity to create a humanized bone‐like tissue in vivo, using tubular mPCL scaffolds seeded with primary osteoprogenitors implanted ortho‐ and ectopically, which allowed more sophisticated bone metastases studies.^[^ ^28^, ^30^ ^]^ Based on these models, the GelMA‐based osteoblast tissue constructs and methodologies presented here led to similar humanized bone formation in vivo, where human cells were maintained and contributed to the bone microenvironment formation. However, in our GelMA‐based model, new bone formation was mainly endochondral, which is more physiologically relevant to bone formation with definitive hematopoiesis,^[^ ^78^ ^]^ yet mPCL scaffolds showed a hybrid endochondral and intramembranous bone formation.^[^ ^30^ ^]^ In summary, here we described a new method of humanized bone tissue formation using GelMA hydrogels cultured only for four weeks in vitro, compared to 6–12 in previous studies, by using a superior mineralization medium before in vivo implantation. This faster approach led to a mature humanized bone tissue similar to a previously well‐established mPCL‐based model. Although both methods led to a mature bone tissue formation, bone marrow content was not derived from the transplanted human cells. This was addressed here with the first humanized fatty bone microenvironment. Many groups have already demonstrated in vivo humanized adipose tissue formation subcutaneously using collagen sponges, silk scaffolds or PEG hydrogels loaded or seeded with human BM‐MSCs, adipose‐derived stem cells or BM‐MSC‐derived adipocytes.^[^ ^79^, ^80^ ^]^ However, to our knowledge, no study showed human adipose tissue formation within a humanized bone microenvironment yet. In the present study, histology analyzes revealed human‐mouse chimeric adipose tissue formation next to the humanized bone tissue after 5 weeks in vivo, proving not only that human adipocytes survived in vivo, but also contributed to the formation of the new adipose tissue. Thus, we described here the first humanized bone microenvironment in rodent containing human adipocytes. The importance of including adipocytes in bone tumor models is only emerging. For the last decade, in vivo studies have demonstrated the potential effect of adipocytes on tumor burden from several cancer types, including prostate cancer.^[^ ^81^, ^82^ ^]^ However, current in vivo models imply murine or human adipocytes interacting with human cancer cells within the rodent bone, and therefore lack species‐specific microenvironment mimicry.^[^ ^7^, ^83^ ^]^ Using our humanized fatty bone model, we not only created the first model to study the effect of human adipocytes on prostate cancer progression within an enhanced species‐specific bone microenvironment, but also confirmed that human adipocytes increased prostate cancer tumor burden.

## 4 Conclusion

We have demonstrated that GelMA‐based hydrogels are a relevant and versatile tool to create bioengineered 3D platforms for mineralized, adipose and tumor microtissue engineering. We have shown that GelMA concentration affected osteoblast mineralization as well as tumor spheroid formation. In addition, we were able to coculture for the first‐time human adipocytes with prostate cancer cells within the same hydrogel, which led to adipocyte dedifferentiation, demonstrating the reciprocal interactions between these cell types. We succeeded in establishing a GelMA‐based humanized bone organ in an animal model, which recapitulated biological functions and morphological features of the human native bone microenvironment, with a shorter in vitro culture time before implantation compared to previous well‐established protocols. This model is the first model of early stage bone tumor formation within the fatty marrow compartment, allowing the analysis of the effects of human adipocytes on prostate cancer bone tumor establishment. The bioengineered humanized model presented here provides a useful tool for the research community by providing a more clinically relevant species‐specific platform. This approach is paramount to improve, in the future, our understanding of human adipocytes and prostate cancer cells interactions in early‐stage bone tumor formation, as well as their contribution to therapy resistance.

## 5 Experimental Section

### Cell Culture

Human primary osteoprogenitors (hOB) were isolated from male donor bone samples, as described previously by our group,^[^ ^30^ ^]^ and approved by the QUT Human Research Ethics Committees (approval number QUT/1400001024). Osteoprogenitors were cultured in osteoblast growth medium (**Table** **1**, all reagent from Gibco, Thermofisher Scientific, Australia). Human BM‐MSCs (ATCC, PCS500012, USA) and human SGBS cells (obtained from Dr. J. Barclay, Mater Research, Australia), were expanded in preadipocyte growth medium (Table 1, all reagent from Gibco, Thermofisher Scientific, Australia). Androgen‐sensitive prostate cancer cell lines LNCaP and C4‐2B, transduced to express luciferase, were a gift from the Australian Prostate Cancer Research Centre Queensland (APCRCQ), under the ethics approval number 1700000184 for work with genetically modified organisms. Both cell lines were cultured in cancer growth medium (Table 1, all reagent from Gibco, Thermofisher Scientific, Australia).

**Table 1:** Cell culture media composition

Media	Components
Osteoblast growth medium	*α*MEM, 10% FBS, 1% P/S
Osteoblastic medium (OM)	*α*MEM, 10% FBS, 1% P/S, 50 ng mL^−1^ ascorbate‐2‐phosphate, 10 × 10^−3^ m *β*‐glycerophosphate, 100 × 10^−9^ m dexamethasone
Mineralization medium (MM)	*α*MEM, 10% FBS, 1% P/S, 100 µg mL^−1^ mOPN, 9 × 10^−3^ m CaCl_2_–2H_2_O, 4.2 × 10^−3^ m K_2_HPO_4_, 25 × 10^−3^ m HEPES
Adipocyte growth medium	DMEM, 10% FBS, 1% P/S, 200 × 10^−3^ m glutamine, 100 mg mL^−1^ heparin, 25 µg mL^−1^ FGF‐1,
Adipogenic induction medium	DMEM, 10% FBS, 1% P/S, 200 × 10^−3^ m glutamine, 100 mg mL^−1^ heparin, 25 µg mL^−1^ FGF‐1, 0.5 × 10^−3^ m IBMX, 0.002 × 10^−3^ m rosiglitazone, 0.25 × 10^−6^ m dexamethasone, 0.02 × 10^−6^ m insulin, 0.1 × 10^−3^ m indomethacin
Adipogenic medium (AM)	DMEM, 10% FBS, 1% P/S, 200 × 10^−3^ m glutamine, 100 mg mL^−1^ heparin, 25 µg mL^−1^ FGF‐1, 0.5 × 10^−3^ m IBMX, 0.25 × 10^−6^ m dexamethasone, 0.02 × 10^−6^ m insulin, 0.1 × 10^−3^ m indomethacin
Cancer growth medium (CM)	RPMI, 5% FBS, 1% P/S
Coculture medium (DMEM)	DMEM, 10% FBS, 1% P/S, 200 × 10^−3^ m glutamine

### Photocrosslinkable GelMA Hydrogels

GelMA hydrogels were prepared using gelatin methacrylamide stock solution (15% w/v porcine Type A, 300 bloom, 80% degree of functionalization, Gelomics, Australia), as described previously.^[^ ^16^, ^25^ ^]^ Briefly, GelMA 4%, 5%, 6%, 7%, and 10% (w/v) hydrogels (5 mm diameter, 2–3 mm high) were prepared by diluting the 15% (w/v) stock solution, previously warmed at 37 °C, with PBS and the photoinitiator (Irgacure 2959; 0.05% (w/v), BASF, Germany). Crosslinking using photoinitiator and 365 nm light has shown no negative effects on cell viability and phenotype and has a protective effect on cells from UV‐generated radicals.^[^ ^84^ ^]^

### Mechanical Testing

Acellular hydrogels were used to determine compressive moduli, or hydrogel stiffness, using Instron 5848 Microtester with a 5 N load cell (Instron, VIC, Australia), as previously described.^[^ ^16^, ^21^ ^]^ Prior to testing, the surface area of each gel was determined using a stereomicroscope and ImageJ software.

### Cell Encapsulation in GelMA Hydrogels

Four different constructs have been used in the study and are described hereafter.

### Human Osteoblast Tissue Constructs (hOTC)

hOBs were used in passages 3–4. After detachment by trypsinization (0.25% Trypsin‐EDTA, Gibco, Thermofisher Scientific), cells were resuspended in 5%, 7%, and 10% (w/v) GelMA precursor solution at the density of 2 × 10^6^ cells mL^−1^. Osteoblast hydrogels were made from 45 µL cell‐loaded precursor solution (2 mm high) and transferred to 48‐well plates. Hydrogels were washed with PBS before culture.

### Human Adipose Tissue Constructs (hATC)

SGBS cells were used in passages 10–13. In parallel, human BM‐MSCs were used in passages 3–5. Cells were resuspended in 4% (w/v) GelMA precursor solution at the density of 2 × 10^6^ cells mL^−1^. Adipose hydrogels were made from 45 µL (in vitro study) to 65 µL (in vivo study) cell‐loaded precursor solution (2–3 mm high) and transferred to 48‐well plates. Hydrogels were washed with PBS before culture.

### Human Prostate Cancer Tissue Constructs (hPCaTC)

LNCaP*‐RFP‐luc* and C4‐2B*‐RFP‐luc* were used in passages 40–42 and 33–35, respectively. Cells were resuspended in 4% or 6% (w/v) GelMA precursor solution at the density of 0.35 × 10^6^ LNCaP cells mL^−1^, and 0.1 × 10^6^ C4‐2B cells mL^−1^. PCa hydrogels were made from 45 µL (in vitro study) to 65 µL (in vivo study) cell‐loaded precursor solution (2–3 mm high) and transferred to 48‐well plates. Hydrogels were washed with PBS before culture.

### Human Adipose and Prostate Cancer Coculture Constructs

Both the SGBS cells and BM‐MSCs were differentiated into adipocytes for three weeks before being stained using DiR solution (5 × 10^−3^ m, Invitrogen, Thermofisher Scientific) for 30 min at 37 °C and resuspended in 4% (w/v) GelMA precursor solution at the density of 2 × 10^6^ cells mL^−1^, with 0.35 × 10^6^ LNCaP cells mL^−1^ or 0.1 × 10^6^ C4‐2B cells mL^−1^. Coculture hydrogels were made from 65 µL cell‐loaded precursor solution each (3 mm high), transferred to 48‐well plates, washed with PBS, and cultured in coculture medium (Table 1).

### Cell Differentiation: Osteoblastic Differentiation and Mineralization

Osteoblast differentiation was induced by osteogenic medium (Table 1). To induce mineralization, a calcium and phosphate supersaturated medium initially described by Thrivikraman et al.,^[^ ^34^ ^]^ also called mineralization medium (Table 1), was used after three days of osteogenic differentiation. Mineralization was induced for three days and replenished every 24 h After mineralization induction, all residual minerals present at the bottom of the wells were carefully removed.

### Adipogenic Differentiation

Adipogenic differentiation started with two days of induction medium, followed by adipogenic medium (Table 1). Rosiglitazone supplements the induction medium only as previous data showed that it might have a negative effect in lipid content in mature adipocytes.^[^ ^85^ ^]^

### Animal Experiments and Monitoring

All animal experiments were conducted under the approval from the University of Queensland Animal ethics Committee (approval number 2021‐AE000353) and in accordance with the *Australian Code of Practice for the Care and Use of Animals for Scientific Purposes*. Four weeks old male NSG mice (strain NOD.Cg‐*Prkdc^scid^IL2rg^tm1Wjl^/SzJ*, Animal Resources Centre, Canning Vale, Western Australia, Australia) were purchased and held at the Biological Resources Facility at the Translational Research Institute (Brisbane, Queensland, Australia). Prior to subcutaneous implantation of one osteoblast construct per flank, the animals were allowed to acclimatize for one week. The hOTCs were embedded in 40 µL of fibrin gel composed of 12.5 µL of human thrombin and 12.5 µL of human fibrinogen (TISSEEL Fibrin Sealant, Baxter Healthcare International), loaded with 22.5 µg of rhBMP‐2 (1.5 µg µL^−1^) (Olympus Biotech Corporation, Hopkinton, MA). Constructs were implanted after polymerization of the fibrin glue. Incisions were closed using vicryl 5–0.16 mm sutures (Team Medical Supplies, NSW, Australia). Mineralized tissue formation occurred in all the hOTCs and was monitored weekly by CT analysis (X‐Cube, Molecubes, Belgium) for six weeks (8 mice, *n* = 16 hOTCs) and quantified using VivoQuant software. After six weeks of bone tissue formation in vivo, hATC (2 mice), hPCaTC (2 mice) and adipocyte/PCa coculture constructs (4 mice) were implanted within the same subcutaneous pocket than the hOTC. Tumor growth was monitored weekly by in vivo bioluminescence imaging (BLI) using a Xenogen IVIS Spectrum (PerkinElmer, Waltham, MA). Intraperitoneal injection of 150 mg kg^−1^ of XenoLight D‐Luciferin Potassium Salt (PerkinElmer) were performed 17 min before image acquisition. At this stage, mineralized tissue formation was monitored fortnightly by CT analysis (X‐Cube, Molecubes, Belgium). At the experimental endpoint, ex vivo BLI was performed on excised humanized tissues and mouse organs within 20 min after luciferin injection. No metastases were observed in the other mouse organs. Therefore, only explanted humanized tissues were fixed in 4% paraformaldehyde (PFA) overnight and stored in ethanol (70%) until further analysis.

### Cell Metabolic Activity

Prestoblue assay (Thermo Fisher Scientific, Waltham, MA, USA) was used to measure metabolic activity. Hydrogels were cultured in 90% culture medium – 10% Prestoblue solution for 3.5 h at 37 °C. Prestoblue solution was transferred in triplicates in 96 well‐plate and fluorescence was measured using FLUOstar Omega (BMG Labtech, Ortenberg, Germany) at ex: 560 nm, em: 590 nm.

### Cell Viability

Fluorescein diacetate (FDA, 1 µg mL^−1^) /propidium iodide (PI, 5 µg mL^−1^) staining solution was used to assess cellular viability in tissue constructs. Hydrogels were washed before imaging (Spectral Spinning Disc Confocal Microscope (Nikon, Minato, TYO, Japan)) with green (ex 488 nm) and red (ex 561 nm) filters. Maximal intensity projections were made from 350 µm thick z‐stacks using 10 µm step size. ImageJ software (version 1.46r, National Institute of Health, USA) was used to quantify cell viability (% living cells to total cells).

### Alkaline Phosphatase Assay

To quantify the alkaline phosphatase (ALP) activity of osteoblasts, hydrogels were collected on day 1, day 14, and day 28 of culture. Media was aspirated, and hydrogels were washed twice with PBS before being transferred into 1.5 mL Eppendorf tubes containing 0.2% Triton‐1X TE buffer and frozen at −80 °C overnight. Hydrogels went under three freeze/thaw procedures (−80 °C/37 °C) before being scraped using 21G needles. Cell lysate was then centrifuged at 10 000 rpm for 10 min at 4 °C. Samples were mixed in a 1:3 dilution in pNPP substrate solution (Sigma‐Aldrich) and protected from light with aluminum foil. After 30 min of incubation at room temperature, the absorbance was measured using a spectrophotometer (Thermo Fisher Scientific) at 405 nm.

### Alizarin Red Staining

For the qualitative staining of mineralization, alizarin red staining (Sigma Aldrich, St Louis, MO, USA) was performed on the osteoblast constructs. Cell‐laden hydrogels were fixed with 100% ice‐cold methanol (Sigma Aldrich, St Louis, MO, USA) for 20 min and washed twice with deionized water. Hydrogels were incubated in 1% alizarin red staining solution (Sigma‐Aldrich) for 20 min. After several washes using deionized water, constructs were air‐dried for 45 min and imaged using phase contrast microscopy (IX73, Olympus, Australia). For semiquantitative analysis, stained constructs were incubated in 10% (v/v) acetic acid solution overnight before being scraped using 21G needles. Mineral oil (Sigma‐Aldrich) was added to the solution and heated for 10 min at 85 °C. Samples were transferred to ice for 10 min and centrifuged 15 min at 20 000 G. Supernatant was neutralized by adding 10% NH_4_OH solution (Sigma‐Aldrich). Samples were transferred in a 96 well‐plate in triplicates and absorbance was measured at 405 nm.

### Osteoimage Mineralization Assay

To detect hydroxyapatite formation in osteoblast constructs, Osteoimage kit (Lonza, Walkersville, USA) was used following the manufacturer's instructions. Spinning Disc Confocal (SDC) images (350 µm thick, 5 µm step size) were taken with the green (ex 488 nm) filter.

### Oil Red O Staining

To visualize lipid droplets formation during adipogenesis, fixed cells were incubated with 60% isopropanol for 15 min before being stain using Oil Red O solution (1 mg mL^−1^, Sigma‐Aldrich, Australia) for one hour. After washes, images were taken using phase contrast microscopy. Image‐based quantification of lipid droplets positive cells were performed using ImageJ analysis software.

### Immunofluorescence

To visualize morphology of adipogenic cells and PCa cells spheroids, constructs were fixed in 4% PFA for 30 min and stained for DAPI (blue, nuclei) and phalloidin (green, F‐actin filament). Briefly, cells were permeabilized using Triton 0.2% (v/v) solution for 10 min before incubation in 0.5% (v/v) BSA (Sigma‐Aldrich) solution for one hour. Constructs were incubated with DAPI (10 µg mL^−1^) and Phalloidin (2.6 µg mL^−1^) overnight at 4 °C. To visualize lipid droplets, adipose constructs were incubated with Nile Red (10 µg mL^−1^) overnight at 4 °C following DAPI/phalloidin staining. Images were taken using a Spectral SDC Microscope with the blue (ex 405 nm), green (ex 488 nm) and red (ex 650 nm) filter sets. Maximal intensity projections were made from 350 µm thick z‐stacks using 10 µm step size.

### Gene Expression Analysis

As described previously,^[^ ^21^ ^]^ bioengineered tissues were incubated in TRIzol reagent (Thermofisher Scientific, Australia) after two washes with PBS (two hydrogels per condition pooled together), and stored at −80 °C. Constructs were mechanically broken using a 21G needle and centrifuged to remove gel material and cell debris. Direct‐zol RNA Miniprep Plus Kit (Zymo Research, USA) was used for RNA isolation following the manufacturer's instructions. SensiFast cDNA Synthesis Kit (Bioline, Australia) was used to reverse transcribe 120 ng of RNA into cDNA. Using QuantStudio 6 Flex system (Applied Biosystems), qRT‐PCR was performed using SYBR Green PCR Master Mix (Applied Biosystems). ΔΔCq method was used to calculate the gene expression, where the geometric mean of the housekeeping genes (7SL and Cyclophilin) was used to normalize the Cq value of each gene. For relative gene expression analyzes, ΔCq were normalized to the ΔCq of BM‐MSC D14 condition. The sequences of all primers used are listed in Table S1 (Supporting Information).

### Adipogenic Cells and Spheroid Size Measurement

Phase‐contrast images of adipogenic cells and PCa cells spheroids were taken weekly. Image‐based quantification of cells, lipid droplets area and spheroid diameter were performed using ImageJ analysis software.

### Histology and Immunohistochemistry

Collected tissues from in vivo study were fixed in 4% PFA overnight, decalcified for ten days in 10% EDTA (pH 7.4), subjected to routine processing and subsequently embedded in paraffin wax. For histology and immunohistochemistry (IHC) analyzes, 5 µm thick sections from the central area of the samples were used. To assess tissue morphology, Hematoxylin and Eosin (H&E) staining was performed using a standard protocol. Safranin O/Fast Green and Masson's trichrome staining were used to characterize bone tissue sections. For Von Kossa staining, sections were stained 5 minutes with silver nitrate (1% w/v, Sigma‐Aldrich) solution and developed 2 minutes in sodium carbonate‐formaldehyde solution (5% w/v). Farmer's Diminisher solution was used to remove unreacted silver. MacNeal's tetrachrome solution (Dorn and Hart Microedge Inc., USA) was used to counterstain sections. For IHC, dewaxed and rehydrated sections were treated as previously described.^[^ ^26^ ^]^ When targeting intracellular proteins, paraffin sections were permeabilized with 0.1% Triton X‐100 in PBS for 6 min. For antigen retrieval, trisodium citrate buffer (pH 6) or 10% EDTA (pH 7.4) were used for either 5 min at 95 °C or 20 min at 80 °C, and proteinase K (Dako, Glostrup, Denmark) for 5–15 min at room temperature. Before incubation with the primary antibody solution (Table S2, Supporting Information), paraffin sections were blocked for 30 min with 2% bovine serum albumin (BSA, Sigma‐Aldrich) or for 10 minutes with Background Sniper (Metagene, Brisbane, QLD, Australia).

### Microcomputed Tomography

Microcomputed tomography (µCT) was used to delineate mineral deposition from embedded osteoblasts on in vitro samples after four weeks of culture, and bone volume fraction on ex vivo samples after 11 weeks of in vivo culture, using SkyScan 1272 µCT (Bruker, USA). Fixed samples were scanned in PBS at 60 kV and 166 µA. Region of interest (ROI) were applied around the tissue on greyscale images and the lower threshold (minimum/maximum: 49/250) was chosen to separate mineralized bone from background noise and was kept constant for every sample. Total volume (TV), bone volume (BV), and bone volume fraction (BV/TV) were measured.

### Statistical Analysis

Three biologically independent in vitro experiments were conducted for all assays and analyzes, unless stated, with two to four technical replicates each. Graphs were generated using GraphPad Prism version 9. Metabolic activity, ALP activity, bone volume and bone density values were normalized to the mean value at day 1 for each condition. mRNA expression levels of adipogenic cells were normalized to housekeeping genes and BM‐MSC day 14 condition, as indicated in the legend of the figure. All data is presented as mean ± standard error mean (SEM). All statistical analyzes were carried out using IBM SPSS Statistics version 2.1 software, using a general linear model (univariate analysis). For all statistical analyzes, significance level was determined as *P < 0.05, **P < 0.001, ***P < 0.001, and ****P < 0.0001.

## Conflict of Interest

D.W.H. is a co‐founder and shareholder of GELOMICS PTY LTD, a Brisbane‐based company developing and distributing hydrogels for 3D cell culture applications. All other authors declare no competing interests.

## Supporting information

Supporting Information

## Data Availability

The data that support the findings of this study are available from the corresponding author upon reasonable request.
