# Supplementary material for: GelMA and Biomimetic Culture Allow the Engineering of Mineralized, Adipose, and Tumor Tissue Human Microenvironments for the Study of Advanced Prostate Cancer In Vitro and In Vivo
Source: Adv Healthc Mater. 2023 Feb 21;12(14):2201701. doi: 10.1002/adhm.202201701 (PMC11469108; doi:10.1002/adhm.202201701)
Supplement: Supplementary file 1 — Supporting Information [file ADHM-12-2201701-s001.pdf]

ADVANCED HEALTHCARE MATERIALS

Supporting Information

for *Adv. Healthcare Mater.*, DOI 10.1002/adhm.202201701

GelMA and Biomimetic Culture Allow the Engineering of Mineralized, Adipose, and Tumor Tissue Human Microenvironments for the Study of Advanced Prostate Cancer In Vitro and In Vivo

*Agathe Bessot, Jennifer Gunter, David Waugh, Judith A. Clements, Dietmar W. Hutmacher, Jacqui McGovern and Nathalie Bock**

Supporting Information

GelMA and Biomimetic Culture Allow the Engineering of Mineralized, Adipose and Tumor Tissue Human Microenvironments for the Study of Advanced Prostate Cancer *In Vitro* and *In Vivo*

*Agathe Bessot, Jennifer Gunter, David Waugh, Judith A. Clements, Dietmar W. Hutmacher, Jacqui McGovern, Nathalie Bock**

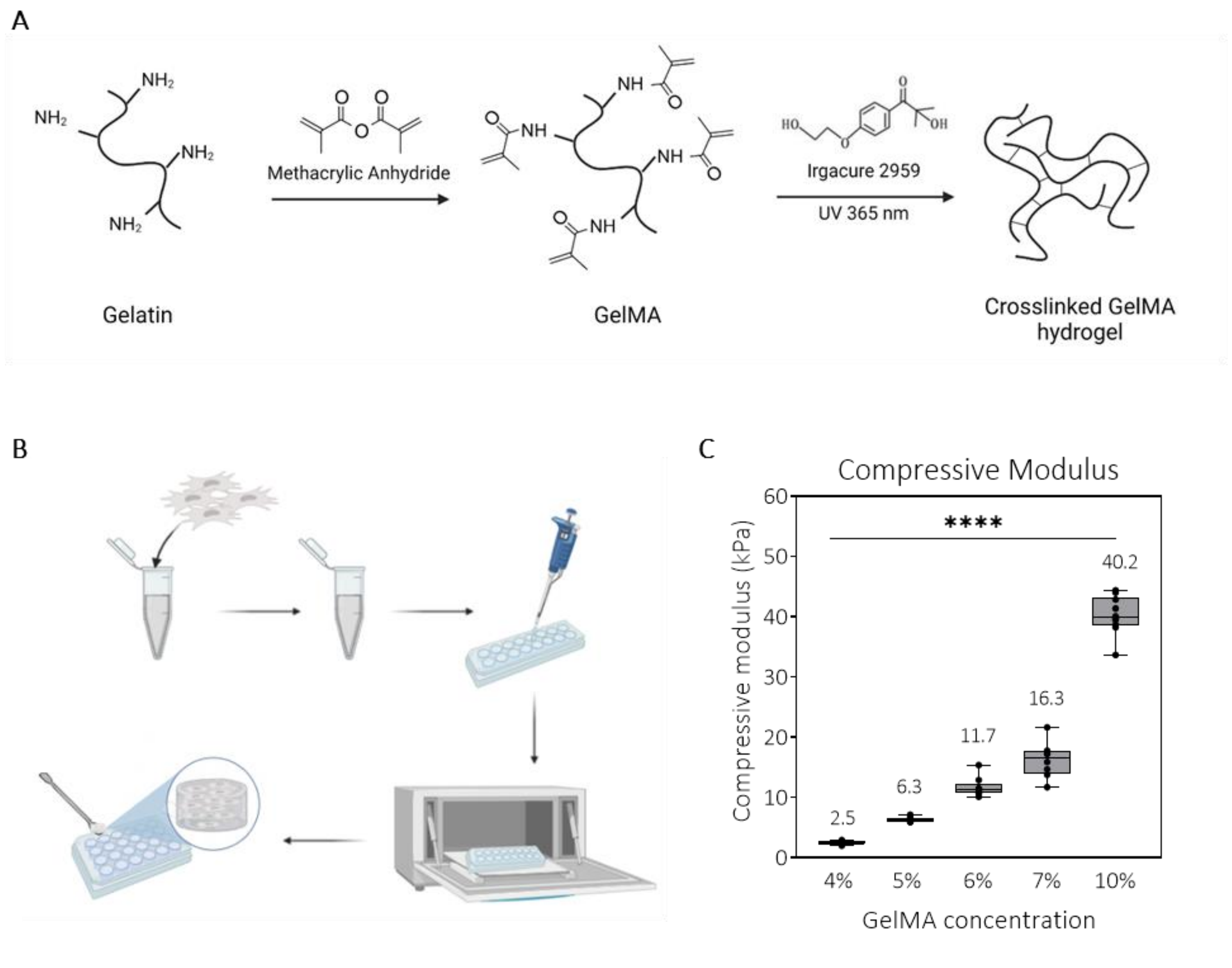

Figure S1. Manufacturing and characterization of GelMA hydrogels. A Synthesis of GelMA hydrogels from gelatin derived with methacrylic anhydride groups and crosslinking using photoinitiator Irgacure 2959 and UV light (365 nm). **B** Cells are suspended in GelMA precursor solution, mixed with photoinitiator Irgacure 2959 and transferred into Teflon molds for 15 minutes of crosslinking at 365 nm. **C** Compressive modulus of hydrogels with GelMA concentration of 4%, 5%, 6%, 7% and 10% showed increase of stiffness correlated with increase of GelMA concentration (n =

9, box and whisker plot (min-to-max), line at median, univariate general linear model, ****P<0.0001).

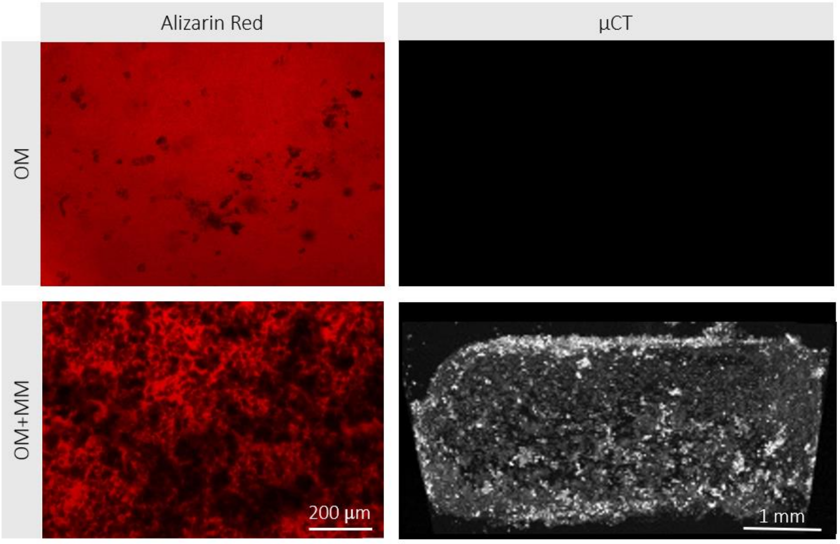

Figure S2. Mineralization medium enhanced faster and higher mineralization compared to standard osteogenic culture. Alizarin red staining and µCT analysis assessed after 4 weeks of osteoprogenitor culture showed higher mineral deposition in culture with mineralization boost (OM+MM) compared to standard osteogenic culture (OM). After only 4 weeks of culture in OM, no minerals could be detected using µCT.

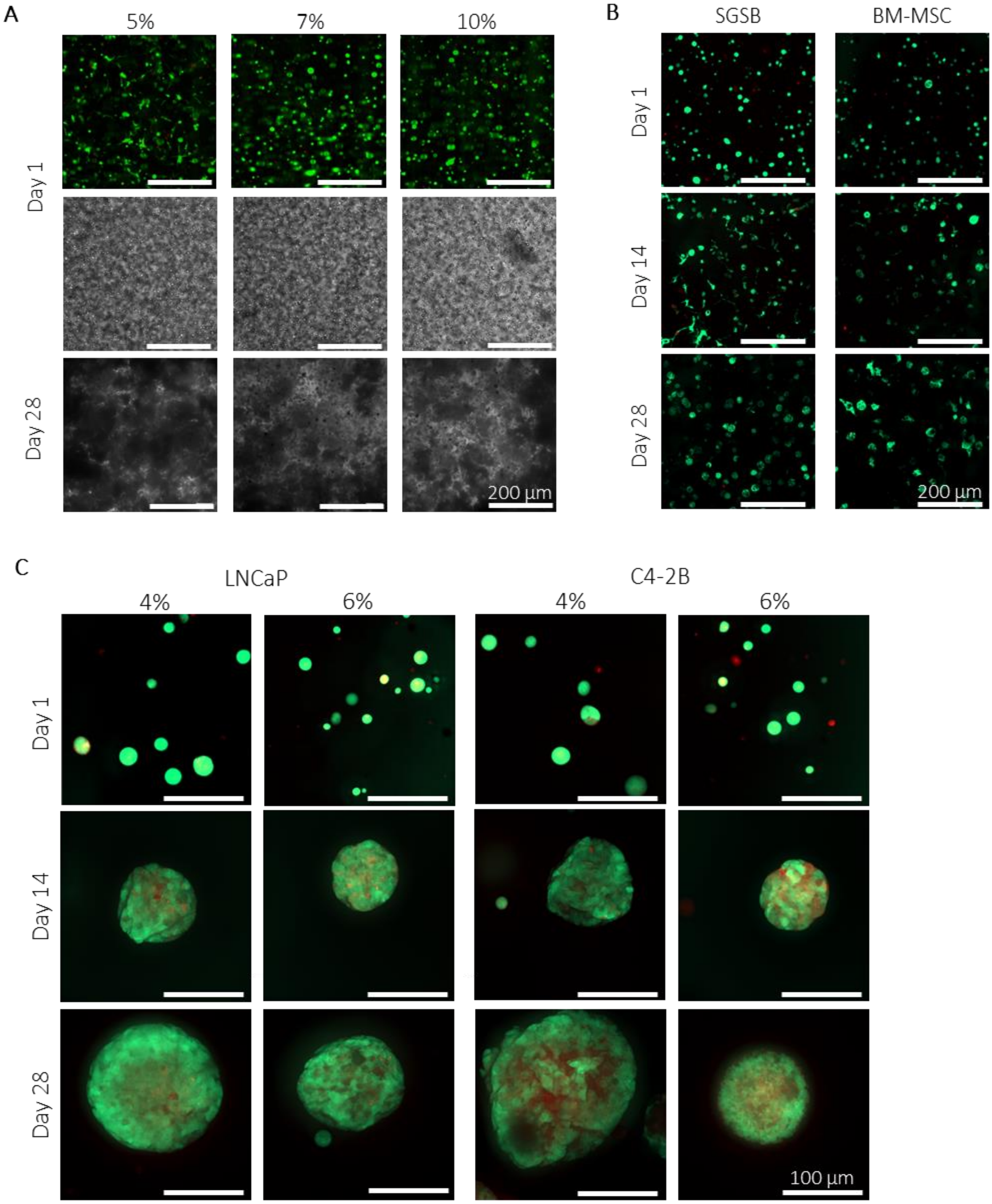

Figure S3. Cell viability of engineered tissue constructs. Cell viability was determined using FDA/PI staining for **A** osteoblast tissue, **B** adipose tissue (Green (FDA): Live, Red (PI): Dead, Scale bar = 200µm) and **C** using FDA staining only for PCa tissue (Green (FDA): Live, Red (GFP): PCa cells, scale bar = 100µm). Each cell type showed a high cell viability over time. Cell viability of **A** osteoblast tissue could not be detected using FDA/PI after 1 week of culture due to deposition of minerals.

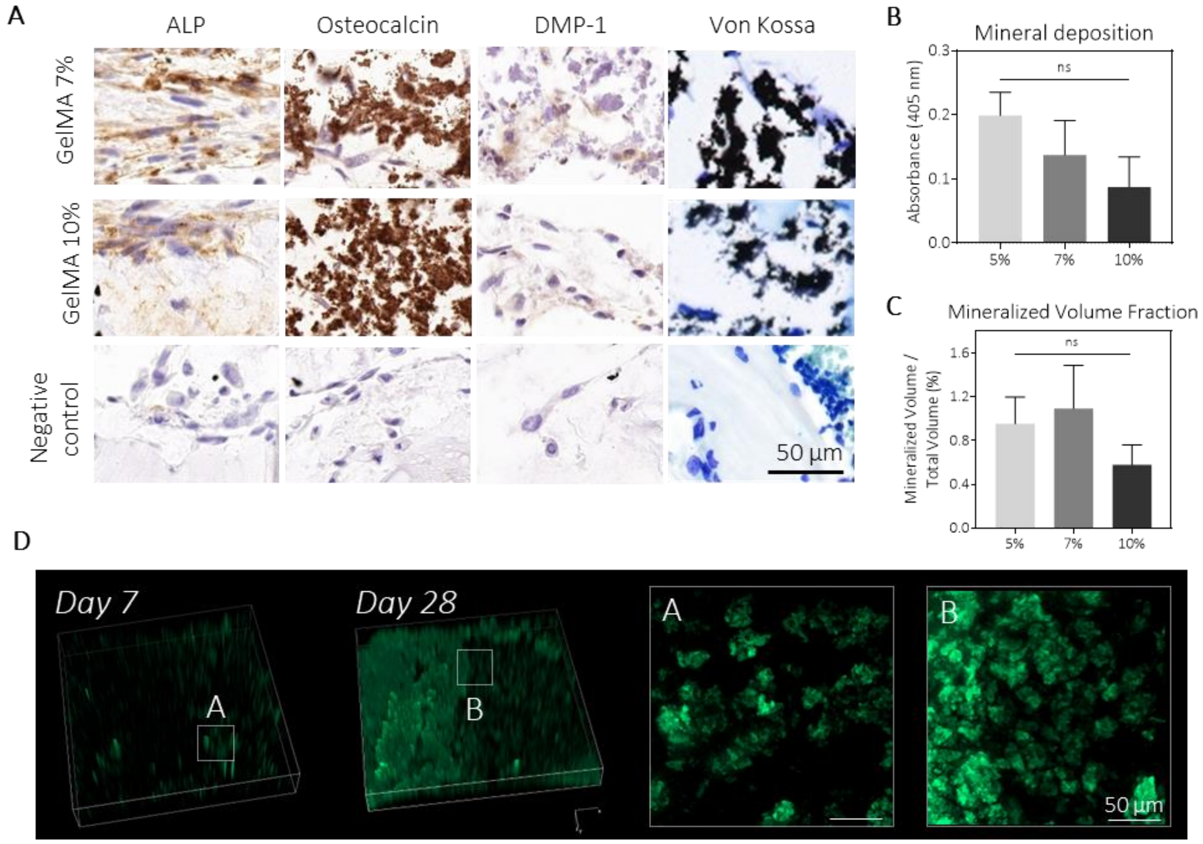

Figure S4. Osteoblast differentiation and minerals detection from osteoblast constructs after a four-week culture period. **A** Representative images of IHC for osteoblastic markers (ALP, osteocalcin and DMP-1) and Von Kossa staining confirm osteoblastic differentiation after four weeks of culture, and minerals deposition (black staining) within the GelMA constructs (scale bar = 50 µm). **B** Alizarin red staining semi-quantification and **C** µCT quantification show mineral content tends to be higher in low stiffness constructs compared to high stiffness, however no significant difference is observed (means ± SEM, n = 3, univariate general linear model, ns = no statistical difference). **D** Representative images of osteoimage staining show hydroxyapatite formation over time (green signal, scale bar = 50 µm).

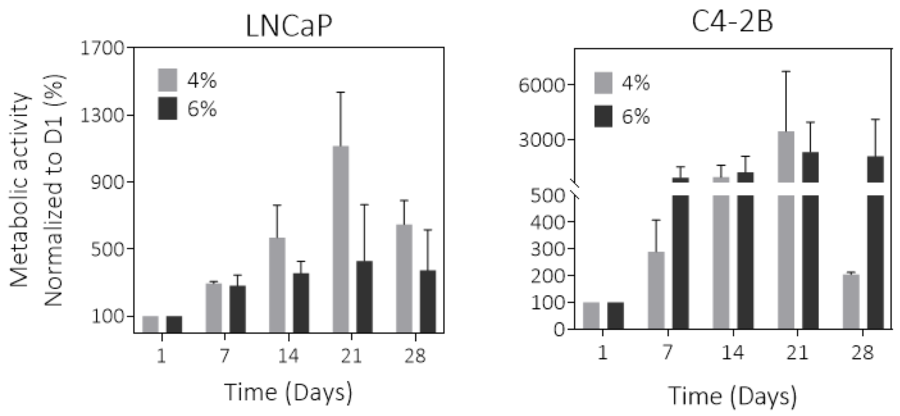

Figure S5. Metabolic activity from prostate cancer tissue constructs. Metabolic activity of PCa constructs shows significant increase of activity over time, for both LNCaP and C4-2B cells, with no significant differences between stiffnesses. Means ± SEM, n = 3, univariate general linear model showed no significant differences.

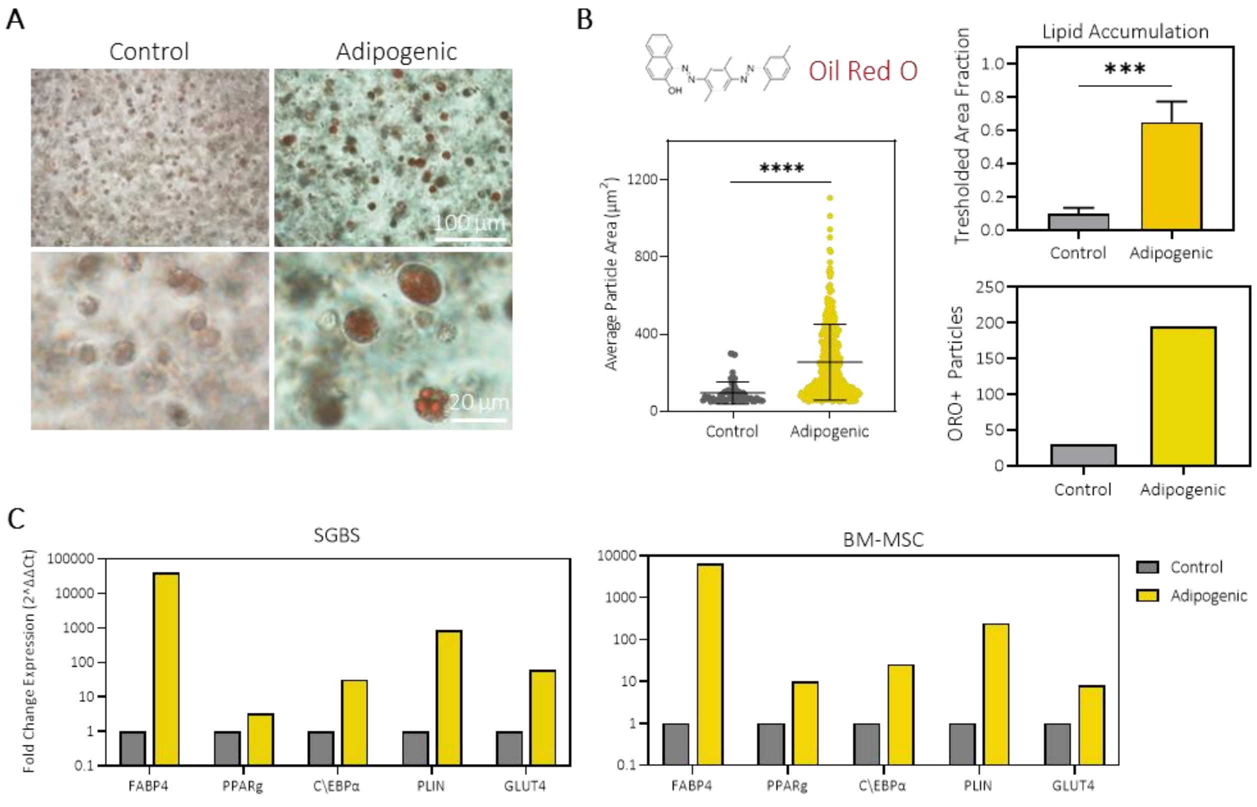

Figure S6. Adipocyte differentiation in GelMA hydrogels after 14 days of adipogenic induction. **A** Brightfield images of Oil Red O staining on SGBS cells shows lipid accumulation in cells cultured in adipogenic medium compared to control (scale bars = 100 and 20 µm). **B** Quantitative analysis of ORO staining from differentiated SGBS cells shows higher lipid accumulation in adipogenic condition compared to control (N = 9 ROI, 226 cells analyzed per condition, mean± SEM, univariate general linear model, ***P<0.001, ****P<0.0001). **C** RT-qPCR analysis of SGBS (left) and BM-MSC (right) shows higher level of adipogenic markers expression in cells adipogenic differentiation compared to control.

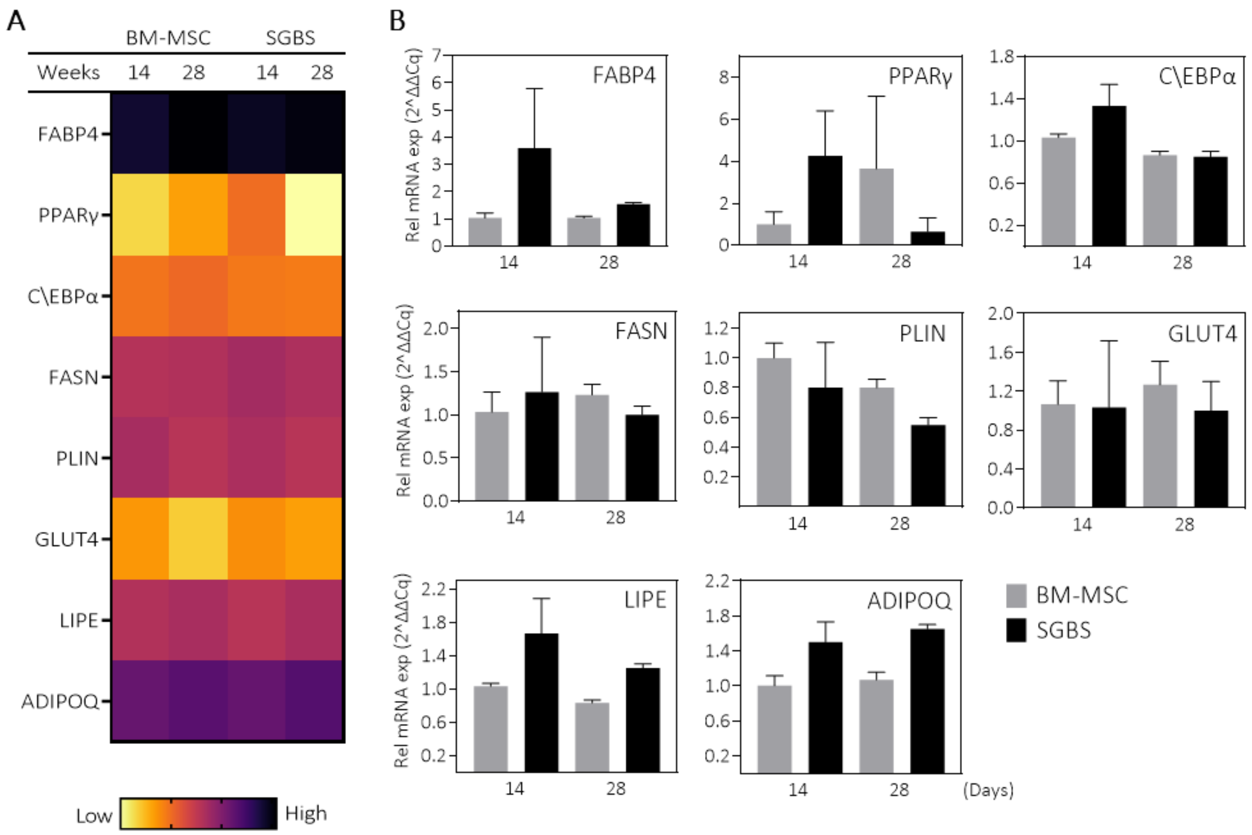

Figure S7. RTqPCR analysis of human adipose tissue constructs after 14 and 28 days of adipogenic differentiation. **A** Heatmap showing *ΔCq* obtained by normalizing *Cq* values to the geomean of housekeeping genes (7SL and Cyclophilin). Yellow and dark purple represent lower mRNA expression (i.e. higher *ΔCq*) and higher mRNA expression (i.e. lower *ΔCq*), respectively. **B** Relative mRNA expression levels in BM-MSC and SGBS cells after 14 and 28 days of adipogenic differentiation. Gene expression levels (*ΔCq*) were normalized to BM-MSC D14 (Means ± SEM, n = 3). Statistical analyses using a univariate general linear model showed no statistical difference between conditions.

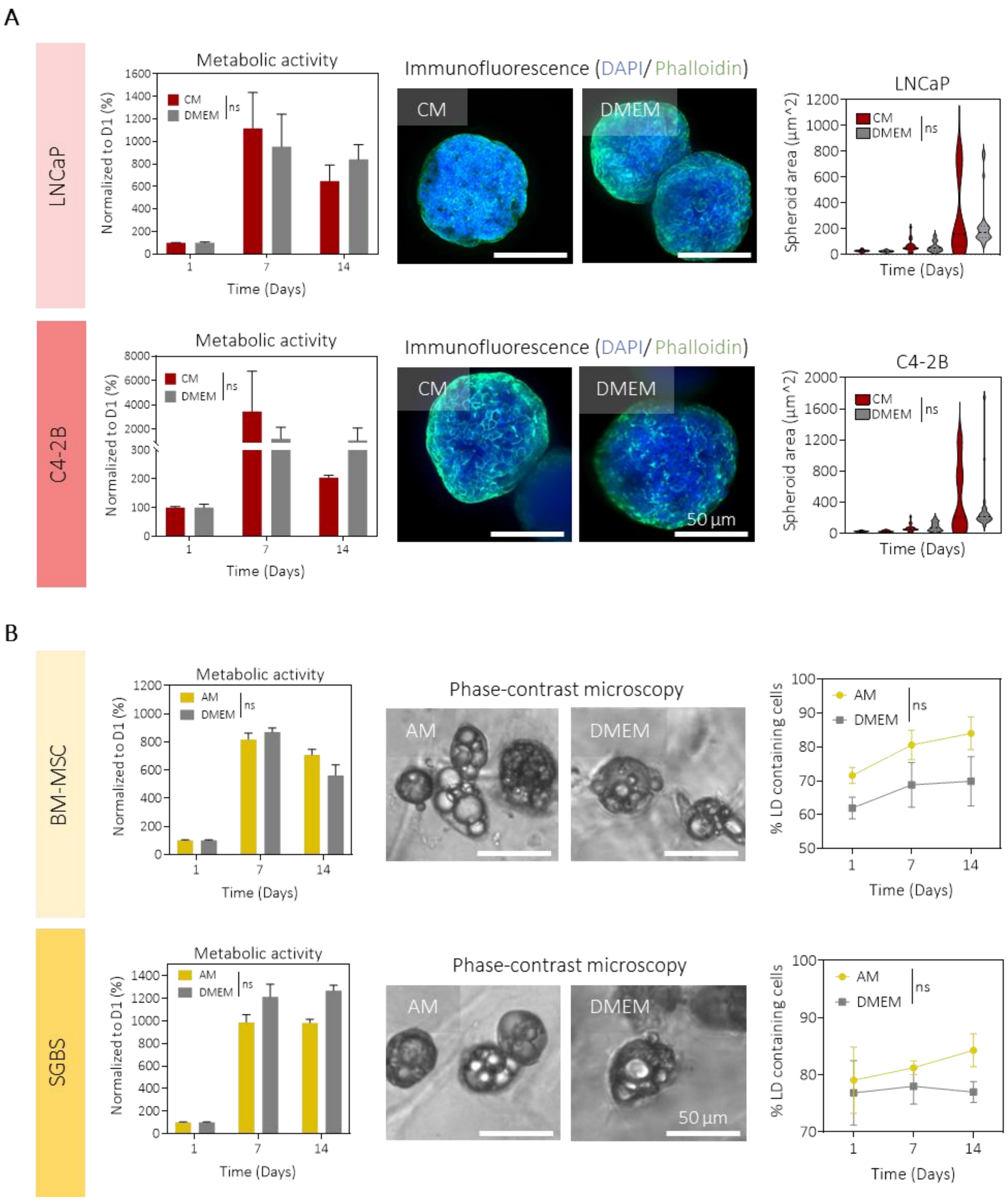

Figure S8. Prostate cancer cells and adipocytes co-culture medium validation on single cultures. A Metabolic activity of prostate cancer cells shows similar metabolic activity between cells cultured in adapted culture medium (CM) and co-culture medium (DMEM). Immunofluorescence for DAPI/Phalloidin images after two weeks of culture (scale bar = 50 µm) and spheroid area quantification shows similar spheroid formation in both culture medium for both PCa cell lines (Violin plots, line at median and quartile, n = 3, N = 80 spheroids analyzed per condition). **B** Metabolic activity of SGBS- and BM-MSC-derived adipocytes shows similar metabolic activity between cells cultured in adipogenic medium (AM) and co-culture medium (DMEM) after 3 weeks of

differentiation prior 3D culture. Representative phase-contrast microscopy images after two weeks of culture (scale bar = 50 µm) and cell quantification shows similar proportion of lipid droplets containing cells over time in both culture medium (N = 84 cells analyzed per condition). Mean ± SEM, n = 3, univariate general linear model, ns = no statistical difference.

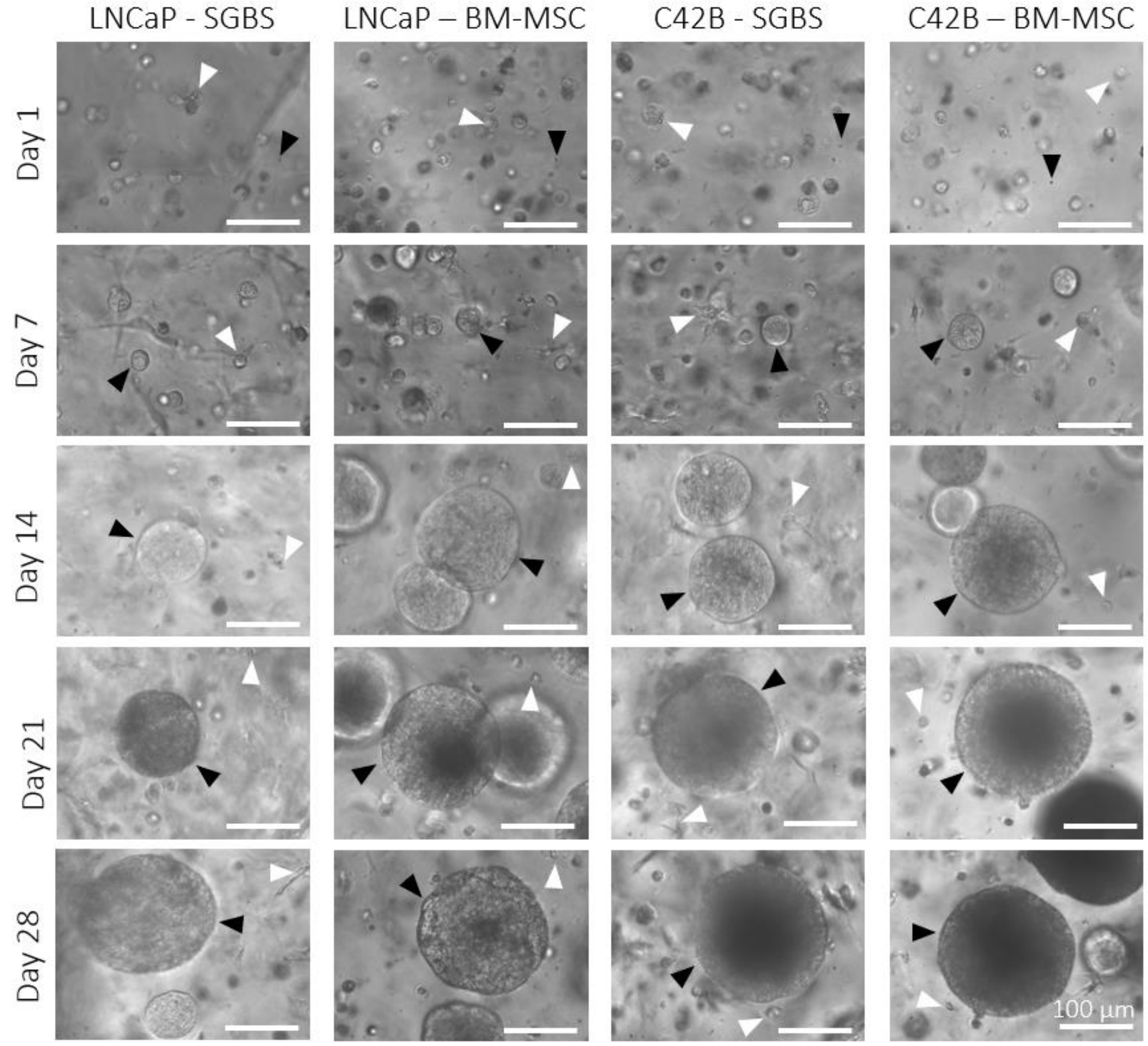

Figure S9. Phase-contrast microscopy images of adipocytes and prostate cancer cells direct co-culture. Representative images showing spheroid formation from LNCaP and C4-2B cells (black arrows) when co-cultured with BM-MSC- and SGBS-derived adipocytes (white arrows) (scale bar = 100 µm).

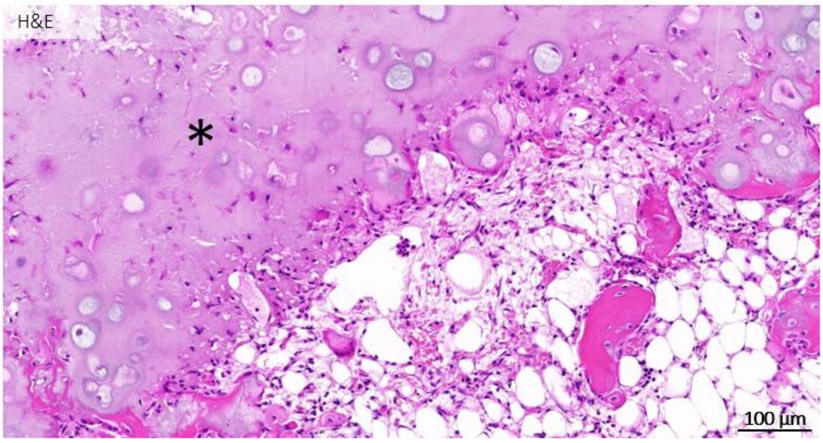

Figure S10. Histological analyses of hOTCs after 11 weeks *in vivo*. H&E staining showed residual GelMA within some hOTCs (black star = residual GelMA, scale bar = 100 µm).

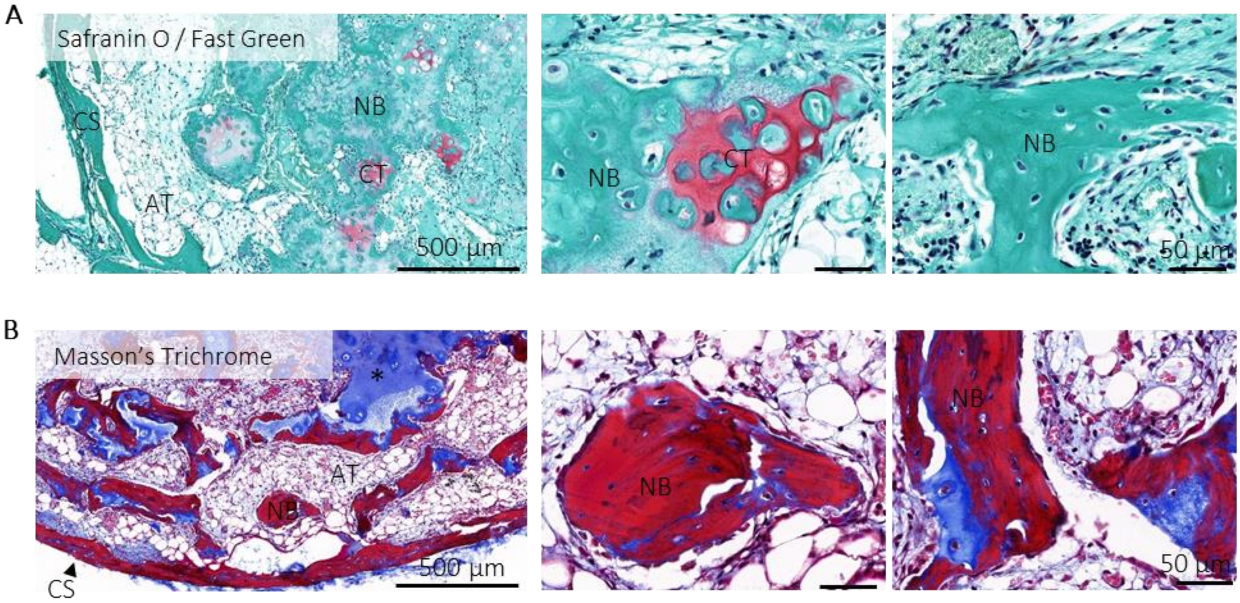

Figure S11. Ossification characterization of hOTCs after 11 weeks *in vivo*. Representative images in low (scale bar = 500 µm) and high (scale bar = 50 µm) magnification of **A** Safranin O/Fast Green staining showing calcified cartilage (red staining) and collagen (green staining) within the hOTCs. **B** Masson's trichrome staining showing newly formed bone (red staining) from collagen deposition (blue staining). Some remaining GelMA was observed in the center of some hOTCs (blue staining indicated with black star). NB, new bone; AT, adipose tissue; CT, cartilaginous tissue; CS, cortical shell.

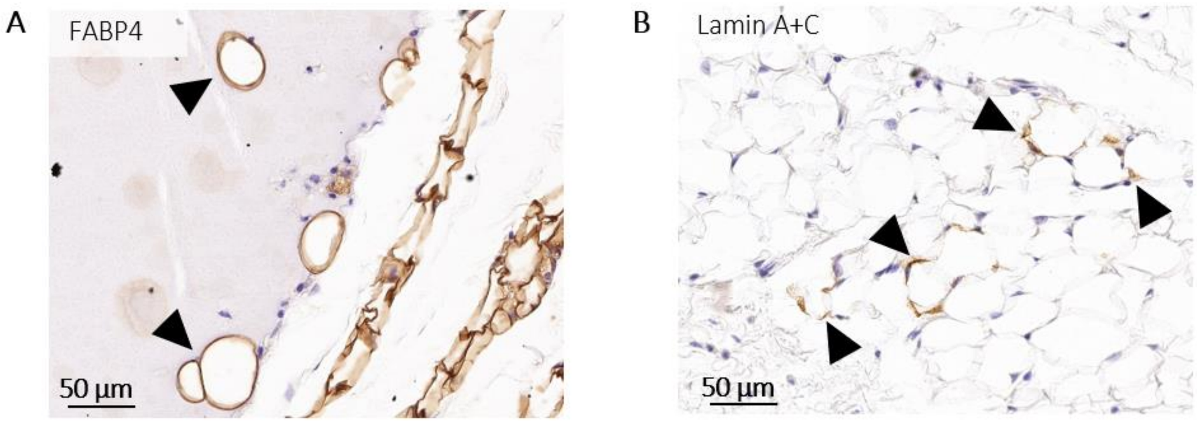

Figure S12. Immunohistochemistry of human adipose microtissues after 5 weeks *in vivo*. IHC for **A** FABP4 showed few adipocytes remaining within the GelMA hydrogel (black arrows). **B** Lamin A+C showed positive cells (black arrows) within the adipose tissue located around the GelMA hydrogel (scale bar = 50 µm).

Table S1. Primers sequences used for RT-qPCR

Target Gene	Primers (5'-3')
**FABP4**	F: GGATGATAAACTGGTGGTGGAATG<br>R: CAGAATGTTGTAGAGTTCAATGCGA
**ADIPOQ**	F: AGTCTCACATCTGGTTGGGG<br>R: CTCTCTGTGCCTCTGGTTCC
**PLIN**	F: ACCCCCCTGAAAAGATTGCTT<br>R: GATGGGAACGCTGATGCTGTT
**LIPE**	F: GCGGATCACACAGAACCTGGAC<br>R: AGCAGGCGGCTTACCCTCAC
**PPARγ**	F: GAAACTTCAAGAGTACCAAAGTG<br>R: AGGCTTATTGTAGAGCTGAGTCTTCTC
**C\EBPα**	F: CCAAGAAGTCGGTGGACAAGAAC<br>R: CACCTTCTGCTGCGTCTCCA
**FASN**	F: CGCTCGGCATGGCTATCT<br>R: CTCGTTGAAGAACGCATCCA
**GLUT4**	F: ATGCTGCTGCCTCCTATGAA<br>R: CAGTTGGTTGAGCGTCCC
**7SL**	F: ATCGGGTGTCCGCACTAAGTT<br>R: CAGCACGGGAGTTTTGACCT
**Cyclophilin**	F: CGCGTCTCCTTTGAGCTGTT<br>R: TCTCCAGTGCTCAGAGCACG

Table S2. Antibodies used for chromogenic immunohistochemical analysis

Protein	Dilution	Cat. No.	Company
**Col-II**	1:200	II-II6B3	DSHB (IA, USA)
**Lamin A+C**	1:300	ab108595	Abcam (Cambridge, UK)
**hCOL-1**	1:300	ab138492	Abcam (Cambridge, UK)
**mCOL-1**	1:300	ab21286	Abcam (Cambridge, UK)
**OCN**	1:500	ab13418	Abcam (Cambridge, UK)
**DMP-1**	1:800	M176	DSHB (IA, USA)
**ALP**	1:100	Ab133559	Abcam (Cambridge, UK)
**FABP4**	1:500	ab92501	Abcam (Cambridge, UK)
**Perilipin-1**	1:250	ab126639	Abcam (Cambridge, UK)
**PSMA**	1:500	ab133579	Abcam (Cambridge, UK)
**PSA**	1:500	A0562	DAKO (QLD, AU)
